# Organic lateral heterostructures with interfacial fluctuation for polarization-resolved photonics

**DOI:** 10.1126/sciadv.aea6228

**Published:** 2026-01-02

**Authors:** Yan-Peng Ye, Qiang Lv, Chao-Fei Xu, Zhao-Ji Lv, Lei Wang, Xue-Dong Wang

**Affiliations:** Institute of Functional Nano and Soft Materials (FUNSOM), Jiangsu Key Laboratory for Carbon-Based Functional Materials and Devices, Soochow University, 199 Ren’ai Road, Suzhou, Jiangsu 215123, PR China.

## Abstract

Organic lateral heterostructures (OLHs) based on two-dimensional (2D) molecular crystals are formed through the coplanar assembly of multiple components, and they hold great promise for optoelectronic applications. However, the precise construction of 2D OLHs with noncoplanar epitaxial layers remains challenging, limiting further exploration of lateral heterojunctions. Here, we present a stepwise vapor-phase growth strategy for synthesizing 2D OLHs with interfacial fluctuation, forming convex, flat, and concave architectures. During the lateral epitaxial growth processes, higher growth temperatures enhance molecular deposition, driving the transition of the secondary component from in-plane to out-of-plane growth. Consequently, the epitaxial layer thickness is precisely tuned from 20 to 800 nm by increasing the growth temperature. The resulting OLHs exhibit orthogonally polarized exciton conversion, with dual photoluminescence emission bands at 450 and 670 nm, functioning as multimode photonic converters. These findings provide a strategy to regulate the epitaxial growth route of 2D OLHs, paving the way for integrated photonic circuits.

## Teaser

Two-dimensional lateral heterostructures with interfacial fluctuation can be used for polarization exciton conversion.

## INTRODUCTION

Two-dimensional (2D) materials have generated considerable research interest in optoelectronics due to their unique structural advantages, including high specific surface area and excellent tunability. For example, 2D materials such as graphene (*1*, *2*), hexagonal boron nitride (*3*, *4*), and rubrene (*5*) have demonstrated immense potential for applications in the development of optoelectronic devices. To date, a large amount of research has focused on the design and synthesis of 2D inorganic crystalline materials (*6*–*8*). Recently, emerging 2D organic crystalline materials have opened up a promising research path in the field of 2D materials research, owing to their designable molecular structure (*9*, *10*), low defect densities (*11*), and controllable optoelectronic properties (*12*–*15*). These unique advantages have powerfully fueled the flourishing development of advanced organic photonics, leading to notable achievements in key technologies such as whispering gallery mode resonators (*16*–*20*), transistors (*21*), and optical waveguides (*22*). For example, Hu and co-workers reported that devices fabricated based on 2D organic crystals with high polarization degree exhibit an anticounterfeiting imaging capability (*23*). Chandrasekar and co-workers have developed a multifunctional and integrated optoelectronic device based on organic crystal, capable of simultaneously performing optical signal reception, wavelength conversion, and transmission (*24*, *25*). However, due to the single chemical composition and limited functions of 2D organic crystals, the future development needs of miniaturization and high integration as the basic building blocks of optoelectronic devices are difficult to meet. Therefore, achieving in-plane and out-of-plane recombination of multiple 2D organic crystals, as well as designing and creating 2D organic heterostructures, is crucial for precisely regulating spatial exciton, photon, and other properties.

Two-dimensional organic lateral heterostructures (OLHs) are constructed through the lateral epitaxy of different 2D organic crystals, which not only combine the unique properties of each component but also use the synergistic interaction between multiple components to produce previously unidentified optical properties (*26*). A unique and underexplored aspect of 2D OLHs lies in the morphology of the in-plane interface between different components. During the fabrication process, the interface between the two components can form three possible types (concave, convex, and flat) with variable heights, enabling precise control of photon transmission based on interface heights. Because of these unique advantages, 2D OLHs have shown remarkable properties in manipulating the transmission and conversion of photons/excitons along two directions and have been applied in various fields such as photonic barcodes (*27*), optical logic gates (*28*), and P-N diodes (*29*). In recent years, coplanar assembly methods of different components based on edge heterogeneous nucleation and in-plane epitaxial growth for the synthesis of 2D OLHs have been widely reported. For example, a coplanar assembly strategy combining vapor/liquid growth techniques has been demonstrated, which has successfully realized OLHs with in-plane integration of two or more materials and applied 2D photon transmission regulation of photonic devices (*30*, *31*). However, the limitation of these strategies for synthesizing 2D OLHs is the single in-plane epitaxial growth mode, which leads to insufficient tunability of the epitaxial layers of OLHs and hinders the further rapid development of OLHs in surface and interface regulation. In addition, the precise photon tuning across distinct interface heights in 2D OLHs with interfacial fluctuation holds critical importance for waveguide transmission optimization. Because of the complexity of the epitaxial growth process, realizing 2D OLHs with noncoplanar epitaxial layers by flexibly adjusting the nucleation sites and epitaxial growth degree of the secondary molecules on seed crystals remains a difficult challenge.

In this work, we propose a stepwise vapor-phase growth strategy for synthesizing 2D OLHs with interfacial fluctuation, which combines 2,6-diphenylanthracene (DPA) and 2,6-bis(4-fluorophenyl)-1,5-dihydroxyanthracene-9,10-dione (DPQ), enabling the fabrication of three distinct types of convex, flat, and concave architectures. The sequential nucleation and growth of two different components are controlled by the differences in their sublimation points. Notably, the molecular deposition process is influenced by the crystal growth temperature. As the temperature rises, the molecular deposition rate accelerates, which drives the transition of the secondary component from in-plane growth to out-of-plane growth. Atomic force microscopy (AFM) studies have revealed that DPA molecules undergo noncoplanar epitaxial growth via screw dislocation behavior. In addition, the heterostructure domains can be finely controlled by adjusting the stoichiometric ratio of the two components. The prepared OLHs enable multichannel output and orthogonally polarized exciton conversion with dual emission bands at 450 and 670 nm, functioning as multimode photonic converters. This study provides innovative ideas for the modulation of noncoplanar epitaxial growth pathways of 2D OLHs and facilitates the development of integrated optoelectronic devices in nontraditional 2D OLHs.

## RESULTS

### Stepwise vapor-phase growth strategy for 2D OLHs

**Fig. 1.**
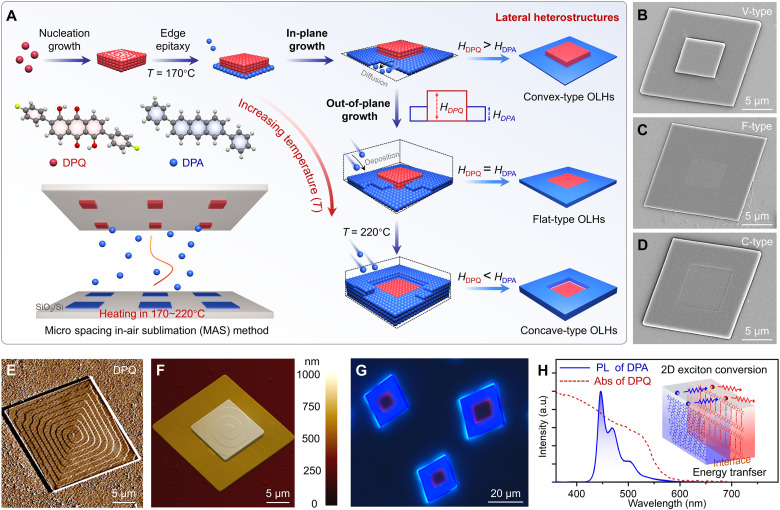
Illustration of the stepwise growth strategy for OLHs. (**A**) Schematic illustrations: a stepwise method for the convex-type, flat-type, and concave-type OLHs. (**B** to **D**) Scanning electron microscopy images of V-type, F-type, and C-type OLHs. (**E**) AFM deflection image depicting the individual DPQ microcrystal under contact-mode operational conditions. (**F**) AFM deflection image depicting the individual V-type lateral heterostructure under contact-mode operational conditions. (**G**) FM image of V-type OLHs. (**H**) PL spectrum of DPA microcrystals and absorption spectra of DPQ microcrystals. a.u., arbitrary unit; Abs, absorption spectra.

DPA molecules and DPQ molecules were selected as the components for constructing the 2D OLHs (figs. S1 and S2). Based on these two materials, we present a stepwise strategy and combine it with the microspacing in-air sublimation (MAS) method (*32*), as shown in Fig. 1A. Three types of 2D OLHs with varying interlayer height differences, which were named convex type (V-type), flat type (F-type), and concave type (C-type), were obtained by controlling the growth temperature of DPA microcrystals. During the crystal growth, both DPA and DPQ molecules self-assemble into rhombic microsheets (figs. S3 and S4). Under ultraviolet light excitation, DPA microcrystals emit blue light with a photoluminescence (PL) peak at 450 nm, and DPQ microcrystals exhibit red emission with a PL peak at 670 nm (fig. S5). The x-ray diffraction (XRD) analysis calculated molecular stacking in DPA and DPQ crystals exhibiting sandwich 2D structures (figs. S6 to S9). The corresponding Commission Internationale de l’Éclairage (CIE) chromaticity coordinate values of DPQ and DPA microcrystals were calculated using their spatially resolved PL spectra to obtain the true luminescence colors of DPQ (0.647, 0.342) and DPA (0.151, 0.089) microsheets (fig. S10).

DPQ was selected as the seed crystal to induce the heterogeneous nucleation and epitaxial growth of DPA because of its stronger thermal stability, which is proven by their thermogravimetric analysis curves (fig. S11). To investigate the suitable temperature range for the growth of OLHs, we conducted growth experiments on single-component DPA and DPQ microcrystals at different temperatures (fig. S12). At a heating temperature of 170°C, DPA molecules sublimated and crystallized on the top substrate, while the DPQ molecules were unable to sublimate due to their greater thermal stability and cannot begin to sublimate and crystallize until the temperature rose to 190°C. Under low-temperature growth conditions, both crystals are weakly luminescent and thin. As the temperature rises, the edge emission of the crystals intensifies. This enhancement can be attributed to the accelerated molecular deposition rate induced by the temperature increase, which promotes an increase in the thickness of the crystals. Therefore, we first prepared DPQ microcrystals as seed crystals. Subsequently, heating the DPA molecules induced their epitaxial growth on the sides of the prepared seed crystals (fig. S13). In general, during the crystal growth process, the deposition rate of molecules is influenced by the growth temperature and follows the Arrhenius equation

$$k=A\;\text{exp}\;\left(-\frac{E_{a}}{\mathrm{RT}}\right)$$

where *A* denotes the pre-exponential factor, *E*_a_ represents the activation energy, *R* is the gas constant, and *T* signifies the absolute temperature. At low temperatures, the rate of molecular deposition is slower, resulting in the epitaxial growth process of DPA being dominated by molecular diffusion. This drives the crystals to undergo in-plane epitaxial growth to form V-type OLHs (Fig. 1B). With the gradual increase in temperature, the rate of molecular deposition is accelerated. The epitaxial growth of the DPA is gradually dominated by molecular deposition. At this stage, DPA gradually transitions from in-plane to out-of-plane epitaxial growth, resulting in the formation of F-type and C-type 2D OLHs (Fig. 1, C and D).

To characterize the growth behavior on the surface of the DPA-DPQ heterostructures, we characterized individual DPQ and DPA microcrystals using AFM (Fig. 1E and fig. S14). A pyramidal growth spiral was found, which continuously developed from the center of the microcrystals to the outer layer. Subsequently, the surface of a DPA-DPQ heterostructure was further characterized through AFM. As shown in Fig. 1F, the noncoplanar epitaxial growth of DPA is realized through screw dislocation behavior. In addition, the fluorescence microscopy (FM) image confirms the existence of V-type 2D OLHs (Fig. 1G). Because of their well-defined structures and optical characteristics, 2D OLHs exhibit distinct band structures and optical properties in different regions, and, thus, excitons can transfer and convert between these regions (*33*, *34*). Notably, DPA microcrystals emit light at 450 nm, which falls within the absorption spectrum range of DPQ microcrystals (Fig. 1H). On the basis of this spectral overlap, the DPA molecules can be excited to singlet excited states using a 405-nm laser. Subsequently, the DPA excitons transfer energy to DPQ molecules in the central region for reabsorption, thereby enabling spatial exciton conversion.

### Structural and optical characterizations of DPA-DPQ 2D OLHs

**Fig. 2.**
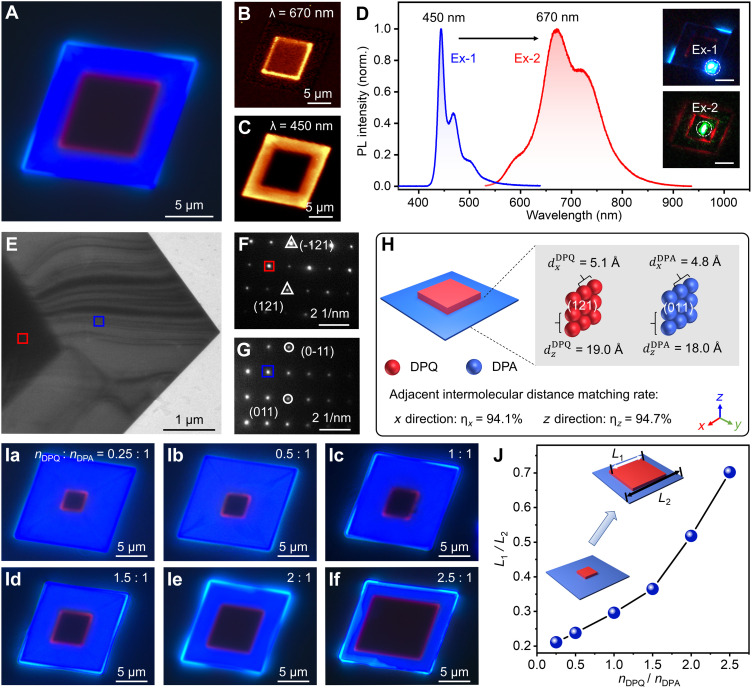
Structural and optical characterizations of V-type OLHs. (**A**) FM image of a V-type DPA-DPQ heterostructure. (**B**) PL map of the V-type heterostructure at 670 nm (red area signal). (**C**) PL map of the V-type heterostructure at 450 nm (blue area signal). (**D**) Corresponding PL spectra collected from the labeled red and blue regions, accompanied by FM images (inset) collected from the V-type heterostructure under excitation at the positions Ex-1 and Ex-2. Scale bars, 5 μm. (**E**) Low-magnification TEM image of a V-type heterostructure. (**F** and **G**) SAED maps of a V-type heterostructure, measured in the red (F) and blue (G) frames in (E). (**H**) The calculation of intermolecular distances matching in two directions on the sides. (**Ia** to **If**) FM images of V-type OLHs with different length ratios; the ratios are 0.21, 0.24, 0.30, 0.37, 0.52, and 0.70, respectively. (**J**) Plot of the lateral ratio (*L*_1_/*L*_2_) versus the stoichiometric ratio of DPQ to DPA. The inset shows a schematic illustration of the V-type heterostructure with tunable blue squares.

By regulating the growth temperature of DPA, three types of DPA-DPQ OLHs with different epitaxial layer thicknesses can be controllably synthesized, named V-type, F-type, and C-type. FM images are shown in Fig. 2A and fig. S15. To verify the distribution of the heterostructure, we conducted PL mapping tests on it using a 405-nm laser. As shown in Fig. 2 (B and C), when 670 and 450 nm were selected, the red and blue regions were separated clearly, confirming the successful synthesis of the lateral heterostructure. Spatially resolved in situ PL spectra obtained from the edge and center regions of the heterostructure contain blue emission from DPA microcrystals and red emission from DPQ microcrystals, indicating the DPA and DPQ components in the heterostructure (Fig. 2D). In addition, the crystalline structure and orientation of DPA-DPQ OLHs were characterized. Simulated crystallographic data showed that DPA [Cambridge Crystallographic Data Center (CCDC) 1044209] and DPQ (CCDC 2435358) crystallize in monoclinic and orthorhombic structures, respectively (table S1). As shown in Fig. 2E, the transmission electron microscopy (TEM) image reveals distinct contrast variations of the DPA-DPQ heterostructure. Specifically, the darker central region and the brighter edge region correspond to the DPQ and DPA structural domains, respectively. Figure 2F presents the selected area electron diffraction (SAED) spots obtained from the central region of the heterostructure, corresponding to be indexed to the (121) plane of the DPQ crystal with d-spacing of 4.96 Å (table S3). The SAED spots from the edge region of the heterostructure can be indexed to the (011) plane of the DPA crystal with d-spacing of 4.76 Å (Fig. 2G and table S2). It was demonstrated that the DPA crystal exhibits a (011) orientation on the (121) plane of the DPQ crystal.

In addition, the mechanisms of DPA heterogeneous nucleation and epitaxial growth were investigated in detail. As shown in Fig. 2H, the adjacent intermolecular distances within the (121) plane of DPQ crystals were calculated using Mercury software. Specifically, the distances along the *x* and *z* directions were found to be 5.1 and 19.0 Å, respectively. Similarly, the adjacent intermolecular distances along the *x* and *z* directions within the (011) plane of DPA crystals were 4.8 and 18.0 Å, respectively (fig. S16). Both the DPQ and DPA crystals adopt a packing method with the long axis of the molecule perpendicular to the substrate, resulting in a high matching rate [η*_x_* = (1 − |5.1 − 4.8|/5.1 = 94.1% and η*_z_* = (1 − |19.0 − 18.0|/19.0) = 94.7%] of intermolecular distances in two lateral directions. Ultimately, this results in the epitaxial growth of DPA on the side of DPQ microcrystals. The XRD patterns of the DPA-DPQ 2D OLHs show distinct diffraction peaks from the (002) planes of DPQ crystals and (100) planes of DPA crystals, confirming the successful synthesis of DPA-DPQ OLHs (fig. S17). To investigate the growth process of the DPA-DPQ OLHs, we used an in situ hot-stage microscopy setup to observe the dynamic morphological evolution in real time. As shown in fig. S18, the DPA molecules continued to grow epitaxially on the sides of the DPQ microcrystal with the gradual increase in heating time. After 6 min, the size of the heterostructure stopped changing. In addition, some transition state images during the growth of DPA-DPQ OLHs were collected by scanning electron microscopy, all of which proved that the OLHs were grown by lateral epitaxy (fig. S19).

Notably, based on the stepwise method for preparing heterostructures, the length ratios (defined as the ratio of red crystalline domain length *L*_1_ to total heterostructure length *L*_2_) of 2D OLHs can be controlled by carefully regulating the stoichiometric ratio of DPQ to DPA (defined as the mole ratio of DPQ to that of DPA). For example, under the same solvent volume conditions, when the stoichiometric ratio of DPQ to DPA was set at 0.25, DPQ microcrystals were first prepared and used as the top substrate for the subsequent growth step. Following this, DPA was heated to sublime, thereby inducing lateral epitaxial growth on the DPQ microcrystals, which resulted in the formation of organic heterostructures with a length ratio of 0.21 on the top substrate (Fig. 2Ia). Keeping the other steps unchanged, adjusting the stoichiometric ratio between the two components to 0.5, 1.0, 1.5, 2.0, and 2.5 sequentially yields OLHs, with corresponding length ratios of 0.24, 0.30, 0.37, 0.52, and 0.70, respectively (Fig. 2, Ib to If). Notably, using the dispersive properties of the solvent, solute molecules (DPQ or DPA) can be uniformly dispersed onto the substrate. Upon heating, these molecules undergo sublimation and subsequently experience uniform nucleation growth or heterogeneous nucleation growth on the top substrate. When the amount of DPQ is increased, the size and number of DPQ crystals also enlarge accordingly. Therefore, if the amount of DPA remains constant, the size and number of seed crystals can be adjusted by increasing or decreasing the amount of DPQ, thereby enabling precise control over the size of the epitaxial layer. In addition, the variation of *L*_1_/*L*_2_ was plotted according to the different molar ratios of the two molecules. As shown in Fig. 2J, there is a positive correlation between *L*_1_/*L*_2_ and stoichiometric ratios of the two components, which proves the high controllability of the stepwise method for preparing the lateral heterostructures.

### Polarized emission properties of DPA-DPQ 2D OLHs

**Fig. 3.**
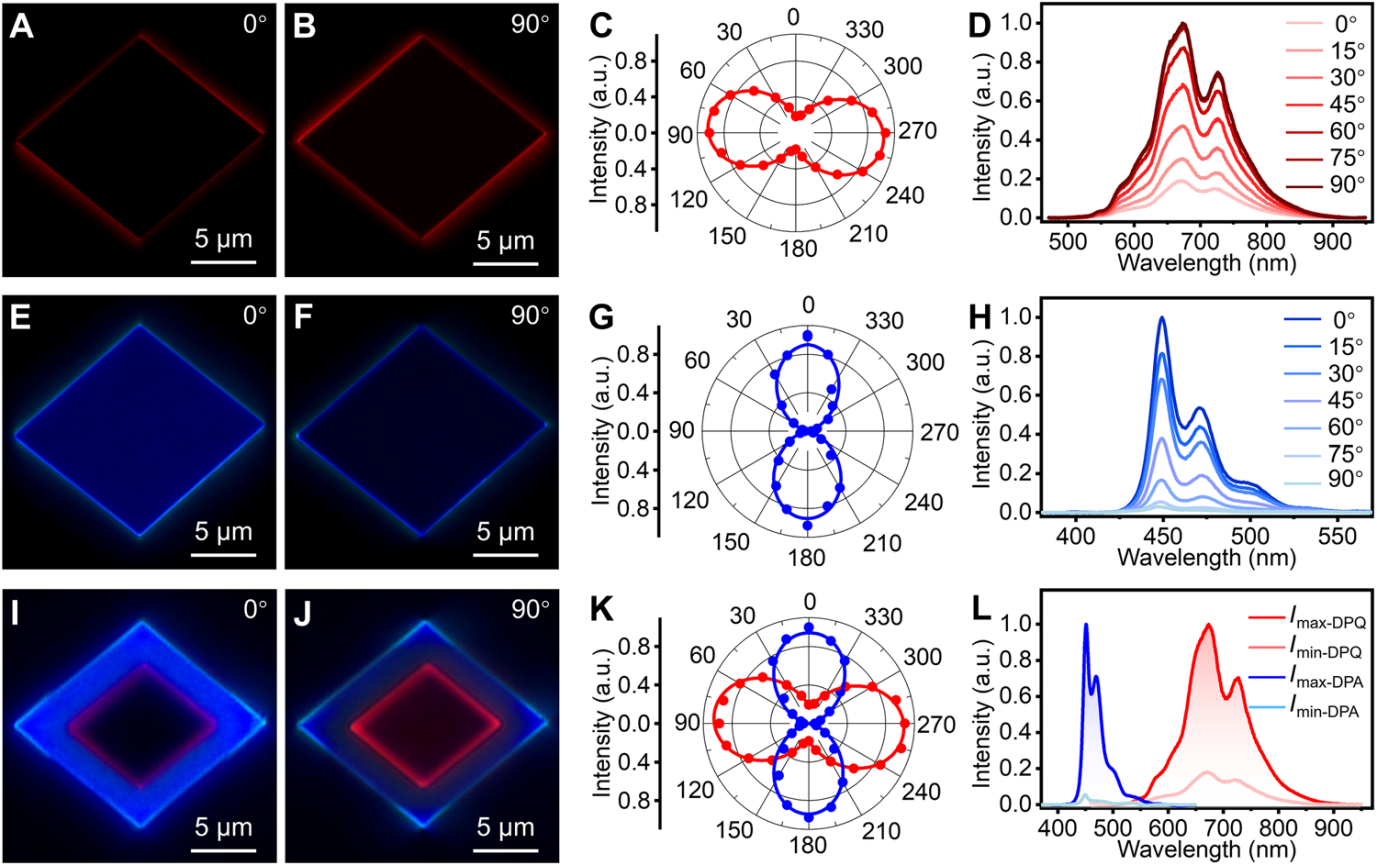
Polarized emission characterization of DPA-DPQ 2D OLHs. (**A** and **B**) FM images of a DPQ microcrystal at polarization angles of 0° and 90°. (**C**) Variation of PL intensity with polarization angle measured from the DPQ microcrystal. The plotted curve is fitted by a sine function. (**D**) PL spectra of the DPQ microcrystal at 0°, 15°, 30°, 45°, 60°, 75°, and 90° polarization angles. (**E** and **F**) FM images of a DPA microcrystal at polarization angles of 0° and 90°. (**G**) Variation of PL intensity with polarization angle measured from the DPA microcrystal. The plotted curve is fitted by a sine function. (**H**) PL spectra of the DPA microcrystal at 0°, 15°, 30°, 45°, 60°, 75°, and 90° polarization angles. (**I** and **J**) FM images of a V-type lateral heterostructure at polarization angles of 0° and 90°. (**K**) Variation of PL intensity with polarization angle measured from the red and blue areas of the V-type lateral heterostructure. The plotted curve is fitted by a sine function. (**L**) The strongest and weakest PL intensities of the red and blue areas of the V-type heterostructure are excited by a 405-nm laser.

The ordered internal molecular arrangement of organic crystals endows them with unique anisotropy and luminescence characteristics (*23*). Molecular arrangements of DPQ and DPA crystals were simulated using Mercury software, whereas the transition dipole moment (TDM) directions of DPQ and DPA crystals were simulated using Multiwfn software (*35*, *36*). As shown in fig. S20, the red and blue arrows represent the TDM directions of DPQ and DPA crystals, respectively. To further investigate the polarized luminescence properties of DPA and DPQ microcrystals, we placed a linear polarizer in the optical path between the sample and the detector. The angle of the linear polarizer is defined as θ. At 15° incremental intervals, FM images of DPQ and DPA microcrystals were captured sequentially. Then, the polarization characteristics of the microcrystals were quantitatively analyzed based on the variation of PL intensity with the polarization angle. As shown in Fig. 3 (A and B), when the angle of the linear polarizer was rotated to 0° or 180°, the DPQ microcrystal exhibited a weak red emission. In contrast, when the angle of the linear polarizer was rotated to 90° or 270°, the DPQ microcrystal exhibited a strong red emission (fig. S21). Notably, the DPA microcrystal exhibited the opposite trend. When the angle of the linear polarizer was rotated to 0° (180°) or 90° (270°), the DPA microcrystal exhibited strong or weak blue emission (Fig. 3, E and F, and fig. S22). The PL intensities of the DPQ and DPA microcrystals were collected at different polarization angles using homemade optical microscopy (fig. S24). As shown in Fig. 3 (C, D, G, and H), the polarization angle-dependent PL intensities demonstrate the anisotropic optical properties of the microcrystals, and the two microcrystals exhibit significant differences in polarization direction in agreement with the calculated TDM direction. Therefore, the polarization properties of a DPA-DPQ heterostructure were analyzed following the same procedure. As shown in Fig. 3 (I and J), when the linear polarizer was rotated to 0° or 180°, the center region of the heterostructure emitted a weak red light, whereas the edge region exhibited a strong blue emission. When the linear polarizer was rotated to 90° or 270°, an opposite trend was observed, featuring enhanced red light emitted in the central region and reduced blue light emitted from the edge region (fig. S23). In addition, the polarization orientations in the central and edge regions of the heterostructure are respectively consistent with those of DPQ and DPA microcrystals (Fig. 3K), which, once again, provides evidence for the successful fabrication of the lateral heterostructure. Figure 3L shows the maximum and minimum values of the corresponding PL intensity in the center and edge regions. These data demonstrate that the synthesis of DPA-DPQ heterostructures has successfully integrated blue and red emissions with nearly orthogonally polarized orientations.

### Controllable thickness of the epitaxial layer in DPA-DPQ 2D OLHs

**Fig. 4.**
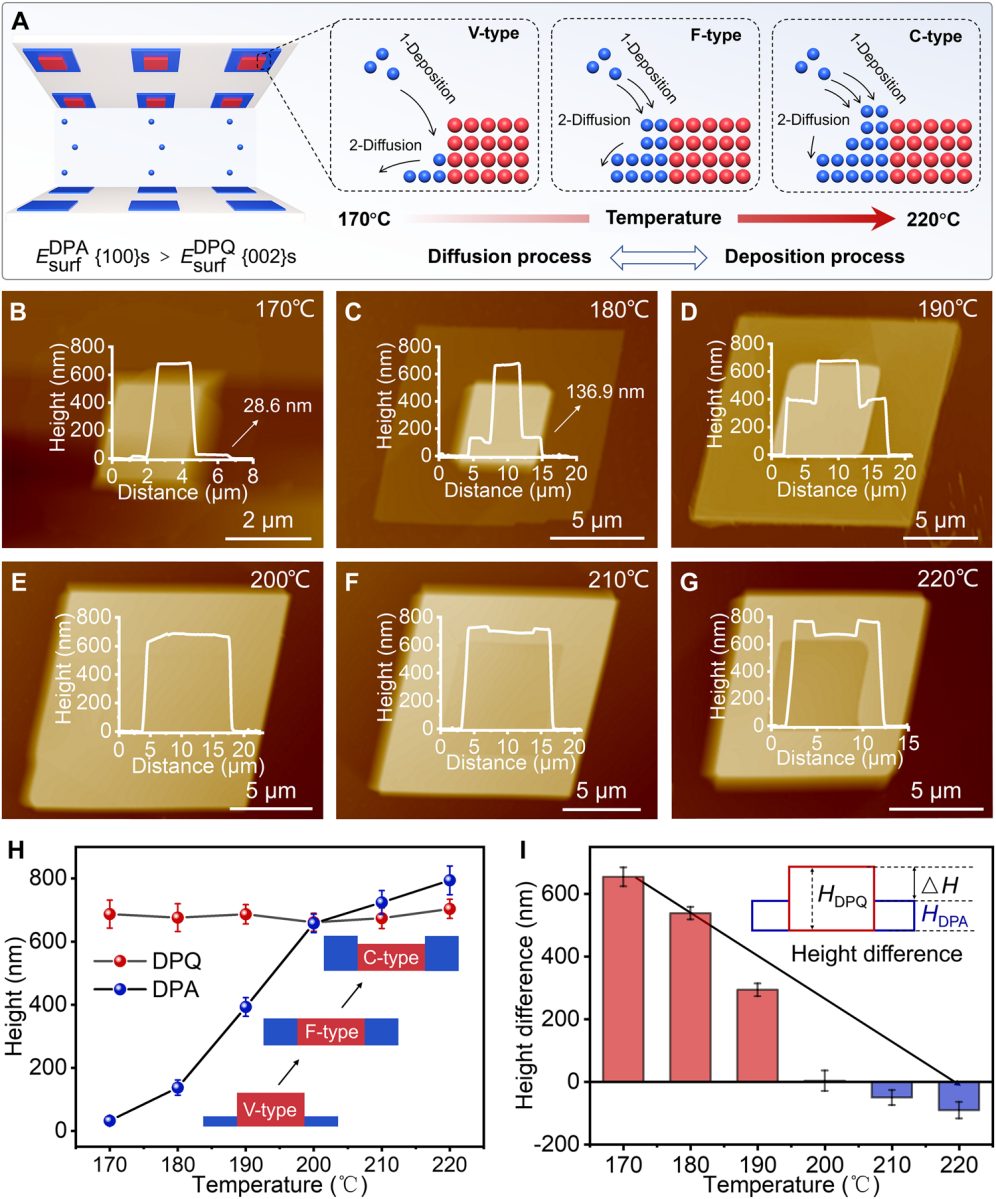
Thickness control of the epitaxial layers in DPA-DPQ 2D OLHs. (**A**) Illustration of the controllable synthesis of DPA-DPQ OLHs with different epitaxial layer thicknesses. (**B** to **G**) AFM height retrace images depicting the DPA-DPQ OLHs at different DPA sublimation temperatures, acquired in contact mode, with (inset) showing the thickness of the DPA-DPQ heterostructures; the temperatures were 170°, 180°, 190°, 200°, 210°, and 220°C, respectively. (**H**) Plot of height of central and edge regions versus DPA heating temperature of DPA-DPQ heterostructures; the number of test samples in each group is 5. The inset shows a schematic illustration of the DPA-DPQ OLHs with tunable epitaxial layer thicknesses. (**I**) Plot of height difference ∆*H* (inset: *H*_DPQ_ is the height of DPQ microcrystals, *H*_DPA_ is the height of DPA microcrystals, and ∆*H* is the height difference between them) versus DPA growth temperature of DPA-DPQ heterostructures; the number of test samples in each group is 5.

To date, precise and tunable synthesis of 2D OLHs with noncoplanar epitaxial layers has rarely been considered, owing to the difficulty of controlling the nucleation sites and epitaxial growth pathways of distinct components. In this work, the epitaxial layer thickness of DPA-DPQ OLHs was controlled by adjusting the growth temperature of DPA, while the thickness of the seed crystals was kept constant. A series of 2D OLHs with different interface areas at the edge surfaces were obtained (Fig. 4A). As shown in Fig. 4 (B and C), when the growth temperatures of DPA were set at 170° and 180°C, the corresponding epitaxial layer thicknesses are 32 ± 14 and 137 ± 24 nm, respectively. As a result, the heterostructures exhibited a convex shape. As the temperatures were raised to 190° and 200°C, the deposition rate of DPA molecules was accelerated, leading to an increase in the thickness of the epitaxial layer to 393 ± 30 and 658 ± 29 nm, and the heterostructures gradually exhibited a flat shape (Fig. 4, D and E). As the temperatures were further increased to 210° and 220°C, the thickness of the epitaxial layer began to surpass that of the seed crystal, reaching 724 ± 38 and 794 ± 45 nm, respectively, which led to the formation of concave heterostructures (Fig. 4, F and G). The corresponding FM images in Fig. 4 (B to G) can be found in fig. S25. Notably, when the thickness of epitaxial layers was comparable to that of seed crystals, the subliming DPA molecules continued to deposit homogeneously on DPA microcrystals rather than on DPQ microcrystals, which was confirmed by the PL mapping of a C-type heterostructure (fig. S26). The reason for this phenomenon is that the surface energy of the DPA (100) plane is higher than that of the DPQ (002) plane (tables S4 and S5). Figure 4H demonstrates that the thickness of DPQ microcrystals remains constant, and the epitaxial layer thickness can be controlled by precisely adjusting the DPA growth temperature. In addition, the interlayer height difference (the height of the seed crystal minus the height of the epitaxial layer) was also calculated. As shown in Fig. 4I, with the increase of the growth temperature of DPA, the height difference of the heterostructures gradually decreased from 655 ± 30 to −90 ± 26 nm, which confirms the tunability of the interlayer height difference in the heterostructures.

### Multiple-output and polarized optical logic gates based on DPA-DPQ 2D OLHs

**Fig. 5.**
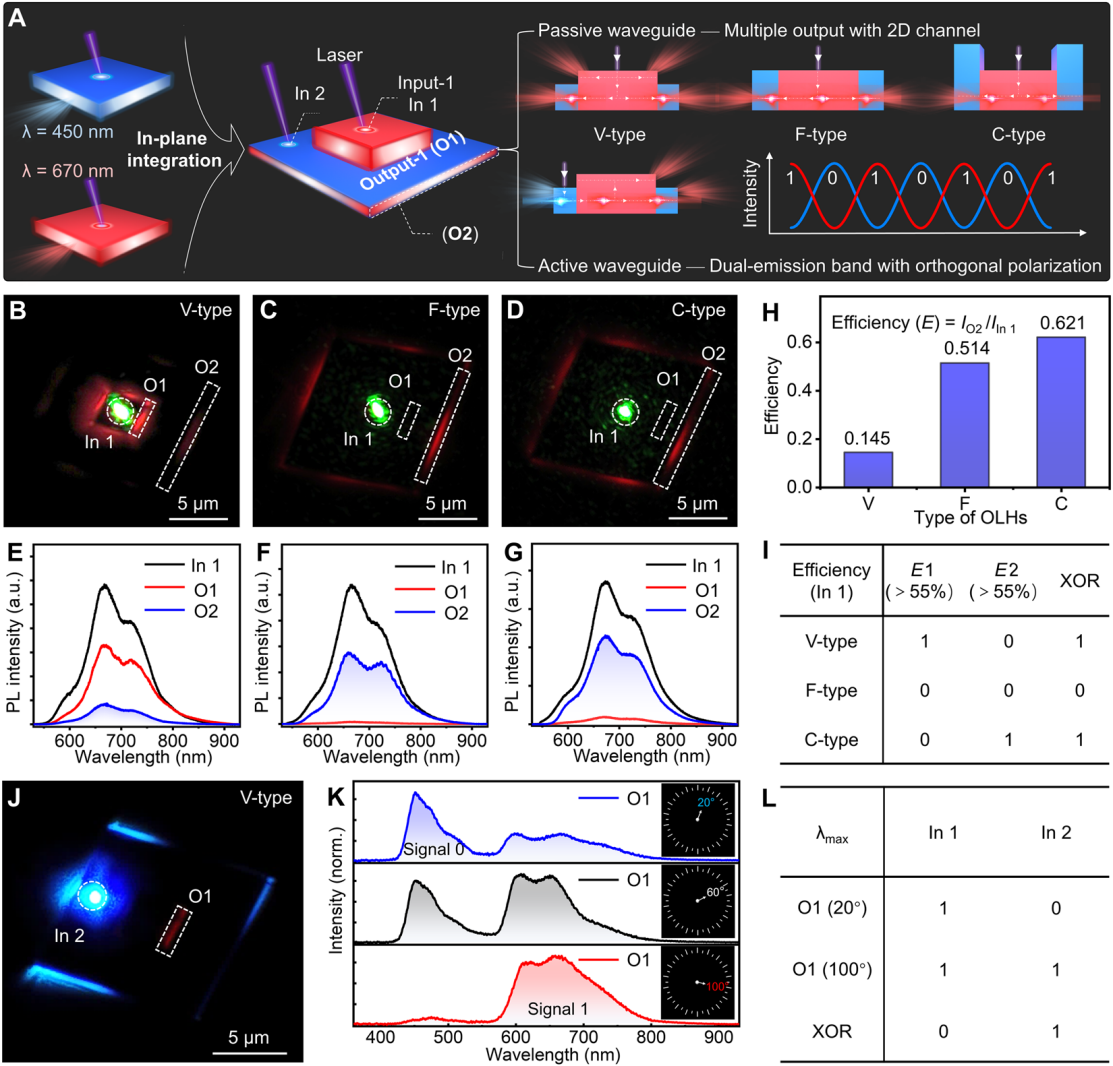
Optical applications of DPA-DPQ 2D OLHs. (**A**) Schematic illustrations of multichannel output and polarized optical signal logic gates based on DPA-DPQ OLHs, along with those of optical waveguides in the edge and central regions of OLHs excited by laser beams. (**B** to **D**) FM images of a 532-nm laser directed at the center area of V-type, F-type, and C-type OLHs. In 1, the laser input excitation point (marked with dashed circles). O1 and O2, laser output edges (marked with white dashed rectangles). (**E** to **G**) PL spectra are obtained from the labeled measurement positions In 1, O1, and O2. (**H**) Plot of output efficiency at the O2 position in V-type, F-type, and C-type OLHs. (**I**) Summary of optical logic tables for three DPA-DPQ heterostructures. (**J**) FM image of a 405-nm laser beam directed at the edge position of the V-type heterostructure. (**K**) Output signals were collected at the O1 position of the V-type heterostructure with corresponding spatially resolved PL spectra at polarization angles of 20°, 60°, and 100°. (**L**) Summary of optical logic tables for the heterostructure at different polarization angles.

Due to their precise structures, 2D OLHs can achieve specific light emission in the edge and central regions. Through adjusting structure and the integration of active/passive optical waveguides, precise control of light transmission can be realized, thus enabling a diverse range of optical functionalities (*37*, *38*). Figure 5A illustrates detailed optical application information based on three types of DPA-DPQ 2D OLHs. Analysis of the optical waveguide properties of DPA and DPQ microcrystals was conducted using the homemade optical microscopy setup. The low optical loss coefficients reveal the excellent optical waveguide capabilities of these two microcrystals (figs. S27 to S30). We selected different excitation positions for the three types of OLHs to facilitate the investigation of photon transport behavior within various regions and across interfaces. As shown in Fig. 5 (B to D), when a 532-nm laser was used to excite the central region of the three types of 2D OLHs, the photon propagated through a passive waveguide mode, resulting in the output signal having a wavelength almost the same as that of the input signal (Fig. 5, E to G). Notably, the PL intensities at O1 and O2 were correlated with the height difference between the layers. In the V-type heterostructure, the PL intensity measured at O1 was higher than that at O2, with output efficiencies *E* (calculated as the ratio of output emission intensity to excitation point emission intensity) of about 56.7% at O1 and 14.5% at O2, exhibiting a dual-channel emission. Conversely, in the F-type heterostructure (i.e., the coplanar heterostructure), owing to the lack of height difference between layers, there was almost no output signal at O1, with output efficiencies of about 51.4% at O2. In the C-type heterostructure, the interlayer height difference reoccurs, leading to an output signal at O1, with output efficiencies of about 5.2% at O1 and 62.1% at O2. As shown in Fig. 5H, with the increase in the thickness of the epitaxial layer, the output efficiency at O2 continuously increases. This can be attributed to the enhanced ability of the epitaxial layer to confine the photons, thereby improving the output efficiency. In addition, the output efficiencies of the three OLHs at the O1 position were also plotted (fig. S31). Based on the correlation between the output position and the output efficiency in three types of OLHs, an exclusive OR (XOR) optical logic gate device was designed. The output efficiency *E* is defined as 1 when exceeding 55%, and 0 otherwise. The truth table for this optical logic gate was summarized in Fig. 5I. Therefore, while the input laser intensity remains, OLHs with various interlayer height differences can be tailored according to the required output intensity of different channels, which facilitates the development of quantitative output of photons in the field of organic photonics.

As already mentioned in Fig. 1H, the emission spectrum of DPA microcrystals overlaps most of the absorption spectrum of DPQ microcrystals; thus, the blue light emitted by DPA microcrystals can be absorbed by DPQ microcrystals. When the 405-nm laser was used to excite the edge part of the V-type heterostructure, photon generated in the DPA region due to excitation propagate through the active waveguide mode, resulting in the emission of red light at the edge of the central part (Fig. 5J). The PL spectrum at the O1 position in Fig. 5J was collected, and it was found that the PL spectrum originating from the DPA part was not completely absorbed by the DPQ (fig. S32). Based on the difference in polarization orientation between DPA and DPQ crystals, the spectra collected at O1 under various polarization angles were measured. At a polarization angle of 20°, the PL intensity of DPA exhibited a higher proportion. As the polarization angle reached 60°, the PL proportions of DPA and DPQ became comparable. Upon further increasing the polarization angle to 100°, it was found that the PL from DPQ demonstrated a higher proportion (Fig. 5K). Using the spatially resolved PL spectra to calculate the CIE chromaticity coordinates of emission colors corresponding to different polarization angles, a color transition from blue to red was displayed (fig. S33). In general, the external coupling signals of 2D photonic logic gates can be realized via exciton conversion and photon propagation (emission and polarization) occurring at the heterojunction interface. In the spectra collected at O1, the maximum value in the emission spectrum range of DPA microcrystals can be defined as the 0 signal, whereas in the emission spectrum range of the DPQ microcrystals, this can be defined as the 1 signal of the XOR logic gate. As a result, it is possible to obtain different logic gate output signals at O1 when the polarization angle is modified. For example, the output signals corresponding to polarization angles of 20° and 100° are 0 and 1, respectively (Fig. 5L). Similarly, the active optical waveguide behavior of F-type and C-type OLHs was also characterized using the homemade optical microscopy setup (figs. S34 and S35). Notably, the current-voltage (*I*-*V*) characteristics of the heterostructure demonstrated bidirectional conductivity (fig S36). Therefore, the prepared 2D OLHs contribute to the realization of multiple output channels and orthogonally polarized exciton conversion, functioning as multimode photonic converters, which facilitate the development of 2D optical logic gate devices.

## DISCUSSION

In summary, we successfully synthesized 2D OLHs with interfacial fluctuation through a stepwise vapor-phase growth strategy, forming three types of convex, flat, and concave architectures. The difference in sublimation points of the two components can be used to control their sequential nucleation and growth. Further investigations revealed that the domain size of heterostructures can be flexibly regulated by fine-tuning the stoichiometric ratio of the two components. In addition, precise control over the thickness of the epitaxial layers was realized by carefully adjusting the growth temperature of DPA to regulate the molecular deposition rate. DPA and DPQ microcrystals exhibited nearly orthogonal polarization orientations. Based on the unique emission and polarization properties of the OLHs, the resulting OLHs can exhibit polarized exciton conversion, functioning as multimode photonic converters. This work presents an advanced strategy for the noncoplanar epitaxial growth pathways of 2D OLHs, facilitating the development of nontraditional 2D OLHs in the field of integrated optoelectronic devices.

## MATERIALS AND METHODS

### Materials

DPA [Chemical Abstracts Service (CAS: 95950-70-2)] was purchased from Tokyo Chemical Industry, 1,5-dihydroxyanthraquinone, iodine (I_2_), iodic acid (HIO_3_), and 1,4-dioxane were purchased from Sigma-Aldrich. Dichloromethane [CH_2_Cl_2_, analytical reagent (AR)] and ethanol (C_2_H_5_OH, AR) were purchased from Aladdin. The quartz substrate was purchased from Suzhou Ketong Bio-pharma. All chemical compounds were directly used without further purification.

### Methods

#### Synthesis route of DPQ

A two-step process was used here: iodination at the 2,6-position and then Suzuki coupling. In a 500-ml round-bottomed flask, a mixture of 1,5-dihydroxyanthraquinone (2.4 g), I_2_ (6.35 g), and HIO_3_ (4.4 g) was dissolved in 1,4-dioxane (200 ml) and H_2_O (40 ml) and refluxed and stirred for 24 hours at 120°C. The resulting solution was then poured into 300 ml of 1 M sodium thiosulfate to quench the reaction and form a precipitate. The solid-liquid was then separated by filtration under reduced pressure, and the resulting precipitate was used directly in the next step. The crude product from the previous step (0.5 g), 4-fluorophenylboronic acid pinacol ester (0.56 g), K_2_CO_3_ (0.6 g), and Pd(PPh_3_)_4_ (0.058 g) were added to a 100-ml Schlenk flask, which was then exchanged three times with argon. Toluene (16 ml), ethanol (EtOH; 4 ml), and water (4 ml) were added under argon atmosphere. The reaction was heated and stirred at 90°C for 24 hours. The reaction mixture was carefully poured into 50 ml of HCl solution (1 M) to form a precipitate, which was then filtered under reduced pressure to give a solid crude product. The crude product obtained was purified by column chromatography [Petroleum ether (PE):dichloromethane (DCM) = 1:1], and the appropriate fractions were concentrated in vacuo to give DPQ as a red solid. The following was used: ^1^H nuclear magnetic resonance (400 MHz, Chloroform-d) δ 13.44 (s, 2H), 7.96 (d, *J* = 7.9 Hz, 2H), 7.76 (d, *J* = 7.9 Hz, 2H), 7.68 (dd, *J* = 8.8, 5.3 Hz, 4H), and 7.19 (t, *J* = 8.7 Hz, 4H).

#### General method of synthesizing DPQ organic microcrystals

First, 2 mg of DPQ was weighed and mixed in 2 ml of DCM and 2 ml of EtOH, and then, 300 μl of DPQ mixed solution was taken and carefully dripped onto a quartz substrate for some time until the solvent was completely evaporated. The substrate was used as the bottom substrate, and a clean quartz substrate was taken as the top substrate, which was continuously heated at 220°C for 6 min, and then, the heating was stopped. The DPQ microcrystals were obtained on the top substrate after the quartz substrate was cooled.

#### General method of synthesizing DPA organic microcrystals

First, 2 mg of DPA was weighed and mixed in 2 ml of DCM and 2 ml of EtOH, and then, 300 μl of DPA mixed solution was taken and carefully dripped onto a quartz substrate for some time until the solvent was completely evaporated. This substrate was used as the bottom substrate, and a clean quartz substrate was taken as the top substrate, which was continuously heated at 190°C for 6 min, and then, the heating was stopped. The DPA microcrystals were obtained on the top substrate after the quartz substrate was cooled.

#### General method of synthesizing DPA-DPQ 2D OLHs

First, 2 mg of DPA was weighed and mixed with 2 ml of DCM and 2 ml of EtOH, and then, 300 μl of the DPA mixed solution was taken and carefully dripped onto a quartz substrate for some time until the solvent was completely evaporated. The substrate was used as the bottom substrate, and the prepared DPQ microcrystals were taken as the top substrate, which was continuously heated at 190°C for 6 min, and then, the heating was stopped. The DPA-DPQ 2D OLHs were obtained on the top substrate after the quartz substrate was cooled.

## Supplementary Materials

### This PDF file includes:

Supplementary Text Figs. S1 to S36 Tables S1 to S5 References
