# Supplementary material for: Organic lateral heterostructures with interfacial fluctuation for polarization-resolved photonics
Source: Sci Adv. 2026 Jan 2;12(1):eaea6228. doi: 10.1126/sciadv.aea6228 (PMC12758536; doi:10.1126/sciadv.aea6228)
Supplement: Supplementary file 1 — Supplementary Text Figs. S1 to S36 Tables S1 to S5 References [file sciadv.aea6228_sm.pdf]

Science Advances
AAAS

# Supplementary Materials for

## Organic lateral heterostructures with interfacial fluctuation for polarization-resolved photonics

Yan-Peng Ye *et al.*

Corresponding author: Xue-Dong Wang, wangxuedong@suda.edu.cn

*Sci. Adv.* **12**, eaea6228 (2026)
DOI: 10.1126/sciadv.aea6228

**This PDF file includes:**

Supplementary Text
Figs. S1 to S36
Tables S1 to S5
References

**Supplementary Text**

Theoretical calculations

The growth morphologies and equilibrium morphology of single crystals were simulated using Materials Studio software based on the attachment energy theory and minimum total surface energy, respectively (*39*). The transition dipole moments of the DPA and DPQ crystals were calculated by the density functional theory (DFT) B3LYP-D3BJ//6-31G(d,p).

Structural and optical characterization

$^{1}$H nuclear magnetic resonance (NMR) spectra were measured on a Bruker 400-MHz spectrometer at 298 K with chemical shifts (δ, ppm) relative to tetramethylsilane ($Me_4Si$). A field emission scanning electron microscope (FESEM, Carl Zeiss, G500) was used to observe the shapes and sizes of DPQ microcrystals, DPA microcrystals, and 2D OLHs. AFM images and HR AFM images were obtained by contact mode of an atomic force microscopy (AFM, Oxford Instruments, Asylum Research, Cypher). Gas molecules were allowed to sublimate and deposit on a carbon-coated copper grid and measured using a TEM (FEI Corporation, Tecnai G2 F20) at room temperature with an accelerating voltage of 100 kV. Bright-field microscopy and fluorescence microscopy (FM) images were obtained using an upright fluorescence microscope (Leica, DM4000 M, Germany) with the sample excited by a mercury lamp. Absorption spectra of organic microcrystals were measured by Lambda 950 (Varian, USA). PL spectra of the microregions were collected using a homemade optical microscope. To measure the concentration spectra of individual crystals, the crystals were locally excited with 405 nm and 532 nm laser, respectively, focused to the diffraction limit. The excitation laser light was filtered through 405 nm and 532 nm notch filters. The light was then coupled to a grating spectrometer (Princeton Instruments, ARCSP-2356) and recorded by a thermoelectrically cooled CCD (Princeton Instruments, PIX-256E). PL spectra were obtained using a confocal Raman imaging system (Alpha300R; 405-nm excitation laser). X-ray diffraction (XRD) patterns were measured by a D/max 2400 X-ray diffractometer using Cu Kα radiation (λ = 1.54050 Å) in the 2θ range of 5° to 30°, with the samples placed on quartz. Single-crystal data were obtained by analysis with Shelxtl software, and CIF files were generated. The molecular structure and conformation were analyzed by importing the corresponding CIF files into Mercury software. The simulated growth morphology and crystal surface parameters were obtained from the single crystal data by the Materials Studio software package.

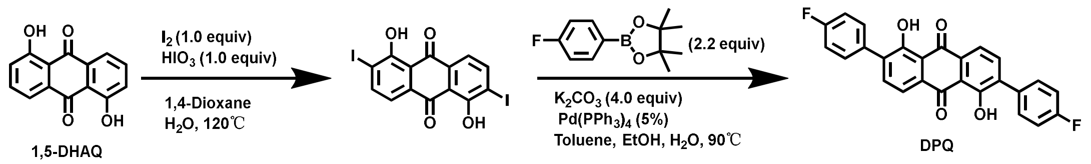


**Fig. S1. Synthetic route of DPQ.**

Iodination at the 2,6-position, then Suzuki coupling.

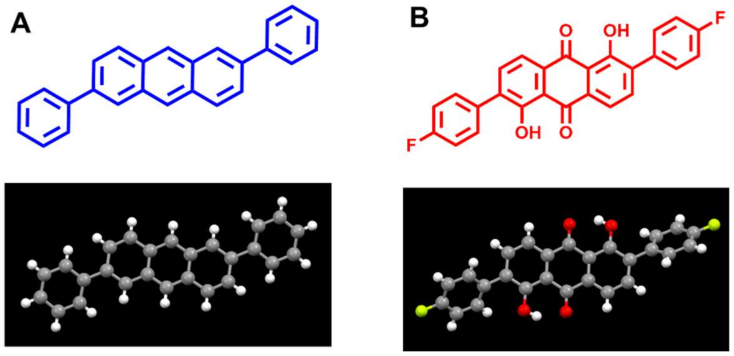


**Fig. S2. Chemical structures.**

Chemical structures of the DPA (**A**) and DPQ (**B**) molecules.

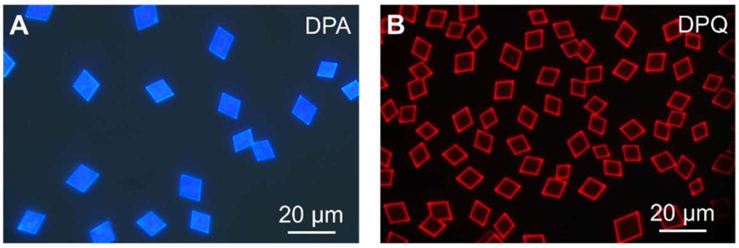


**Fig. S3. Crystal morphology.**

Fluorescence microscopy (FM) images of DPA (**A**) and DPQ (**B**) microcrystals.

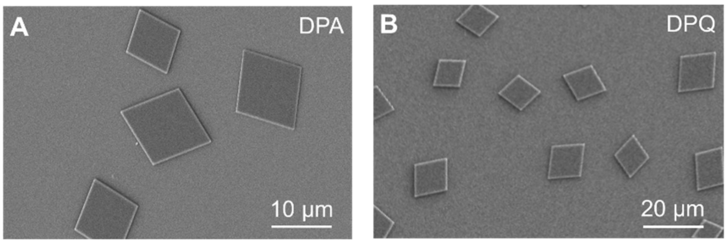


**Fig. S4. Morphology characterizations.**

Scanning electron microscopy (SEM) images of DPA (**A**) and DPQ (**B**) microcrystals.

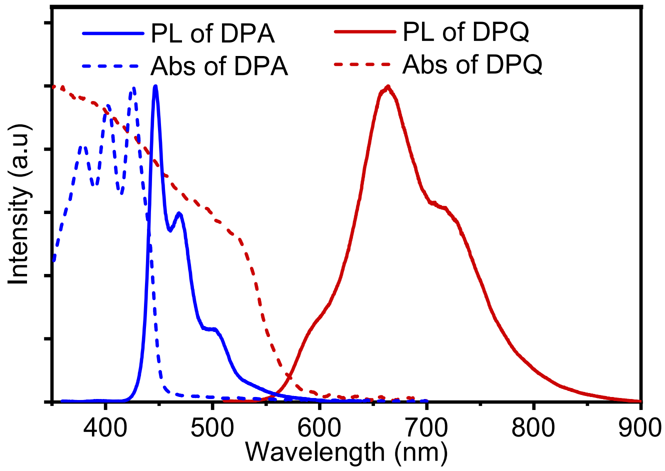


**Fig. S5. Normalized absorption and photoluminescence (PL) spectra.**

Absorption and PL spectra of DPA (blue curve) and DPQ (red curve) microcrystals.

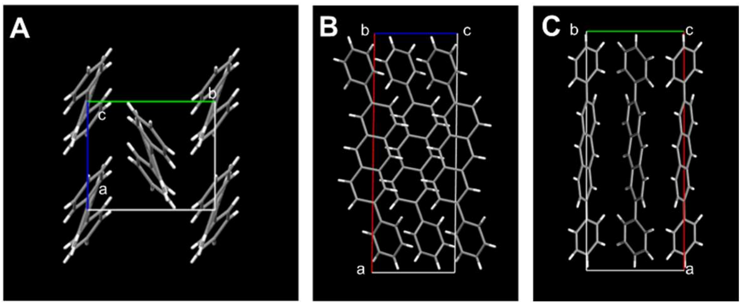


**Fig. S6. Molecular packing structures of DPA crystals.**

Single crystal packing of DPA view along a direction (**A**), view along b direction (**B**), and view along c direction (**C**).

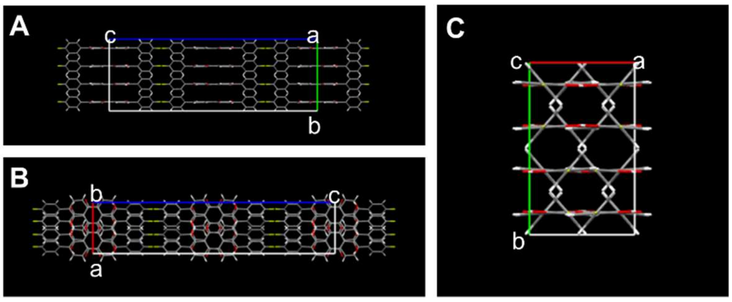


**Fig. S7. Molecular packing structures of DPQ crystals.**

Single crystal packing of DPQ view along a direction (**A**), view along b direction (**B**), and view along c direction (**C**).

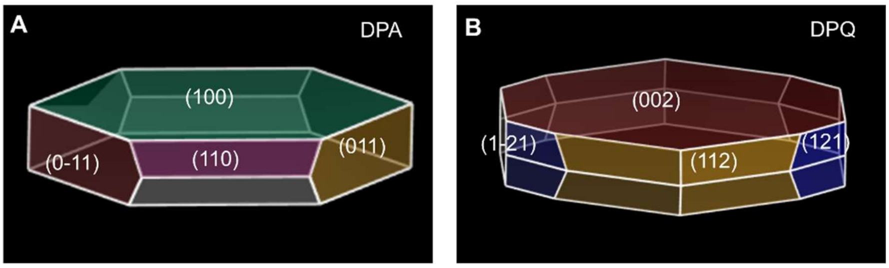


**Fig. S8. Predicted growth morphologies.**

Predicted growth morphology of DPA (**A**) and DPQ (**B**) single crystals based on attachment energy calculated using Materials Studio software simulations.

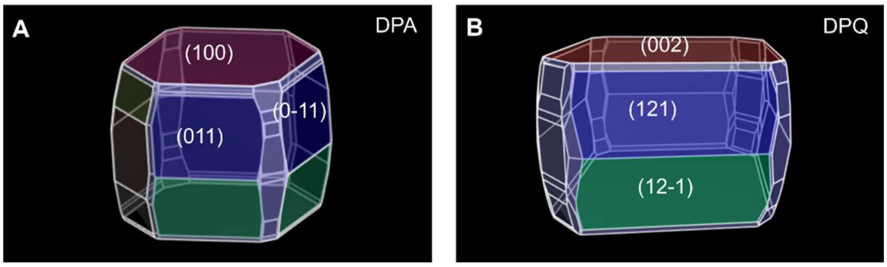


**Fig. S9. Predicted equilibrium morphologies.**

Predicted equilibrium morphology of DPA (**A**) and DPQ (**B**) single crystals based on minimum total surface energy using Materials Studio software simulations.

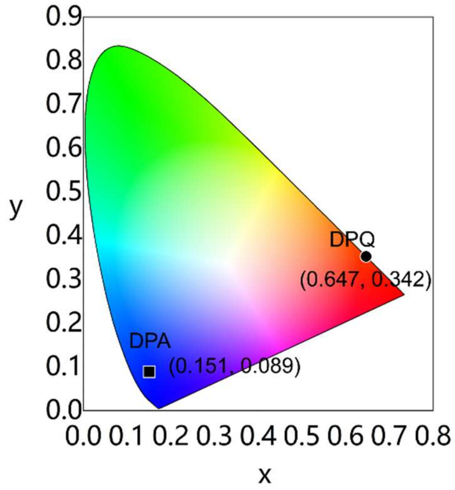


**Fig. S10. Optical properties of the DPA and DPQ microcrystals.**

Commission Internationale de l'Eclairage (CIE) chromaticity diagram corresponding to the DPA and DPQ microcrystals.

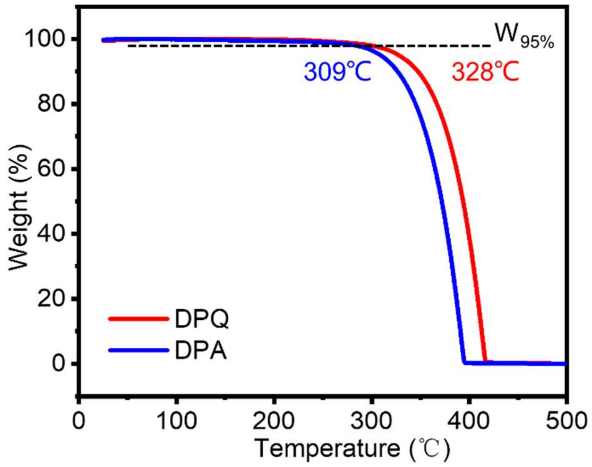


**Fig. S11. Thermal stability analysis.**

Thermogravimetric analysis curves of the DPA (blue curve) and DPQ (red curve) powders in air atmosphere.

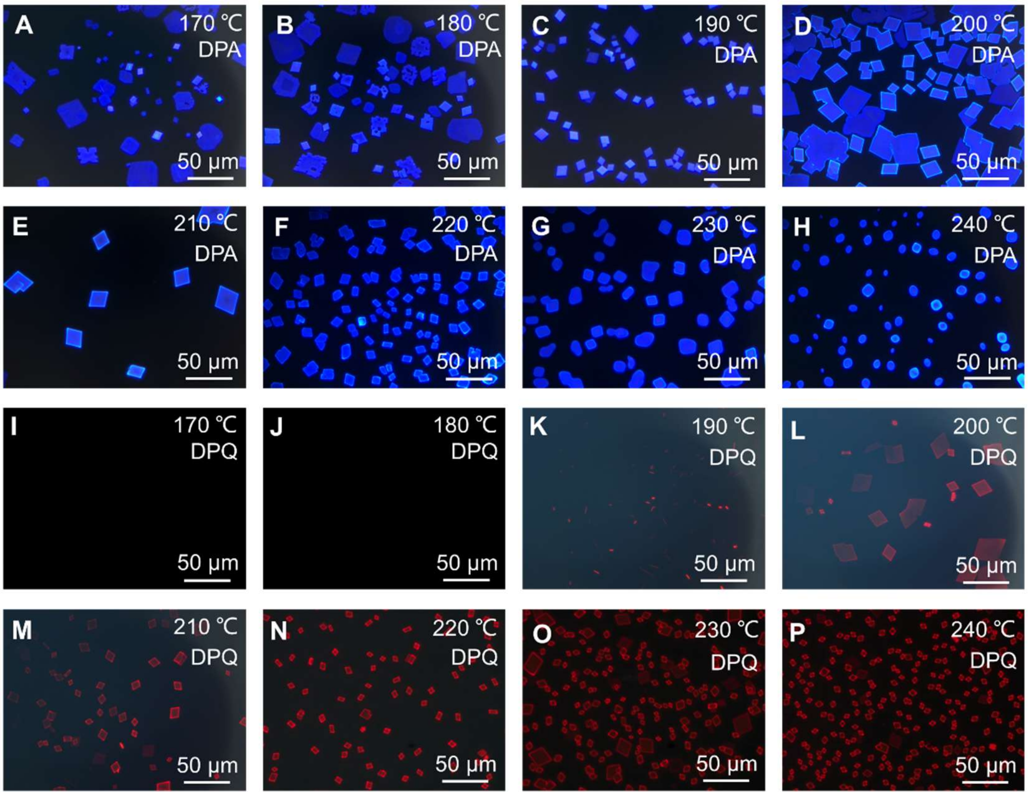


**Fig. S12. Growth temperature analysis experiment.**

FM images of DPA (**A**-**H**) and DPQ (**I**-**P**) microcrystals prepared by the microspacing in-air sublimation (MAS) method at different sublimation temperatures.

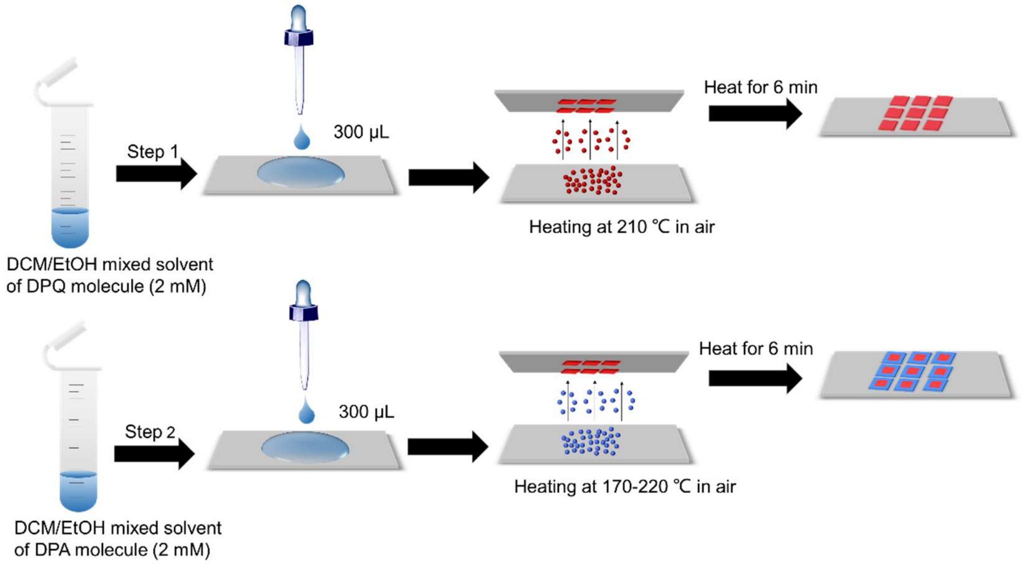


**Fig. S13. Illustration of the process of preparing heterostructures.**

Stepwise method for the synthesis of DPA-DPQ OLHs by the MAS method.

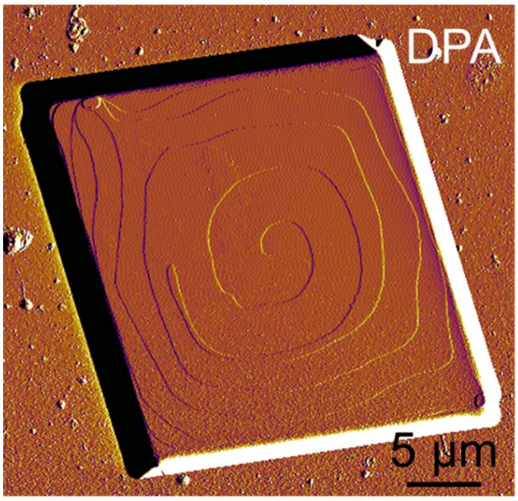


**Fig. S14. Morphology characterization.**

AFM deflection image of a single DPA microcrystal in contact mode.

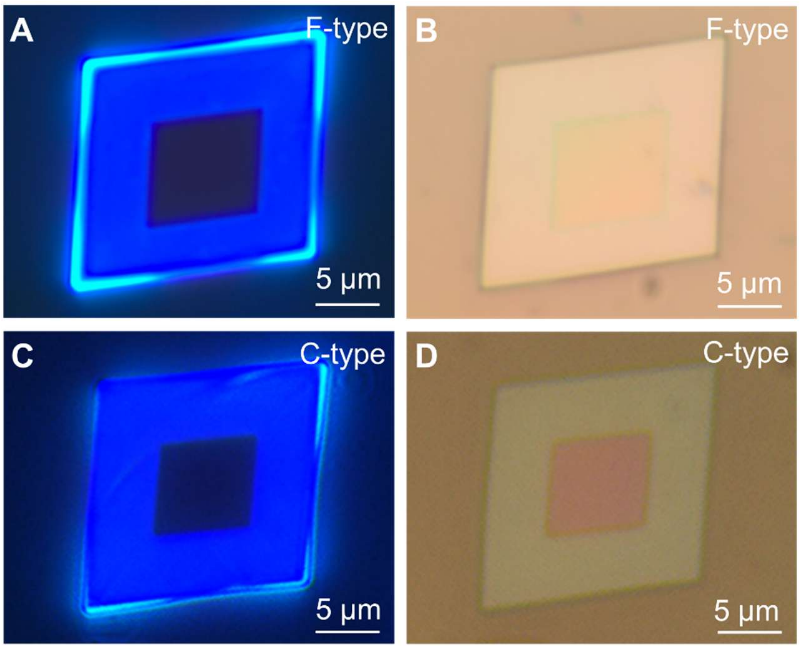


**Fig. S15. Heterostructures morphology.**

FM (**A**) and bright-field microscope (**B**) images of a single F-type DPA-DPQ lateral heterostructure. FM (**C**) and bright-field microscope (**D**) images of a single C-type DPA-DPQ lateral heterostructure.

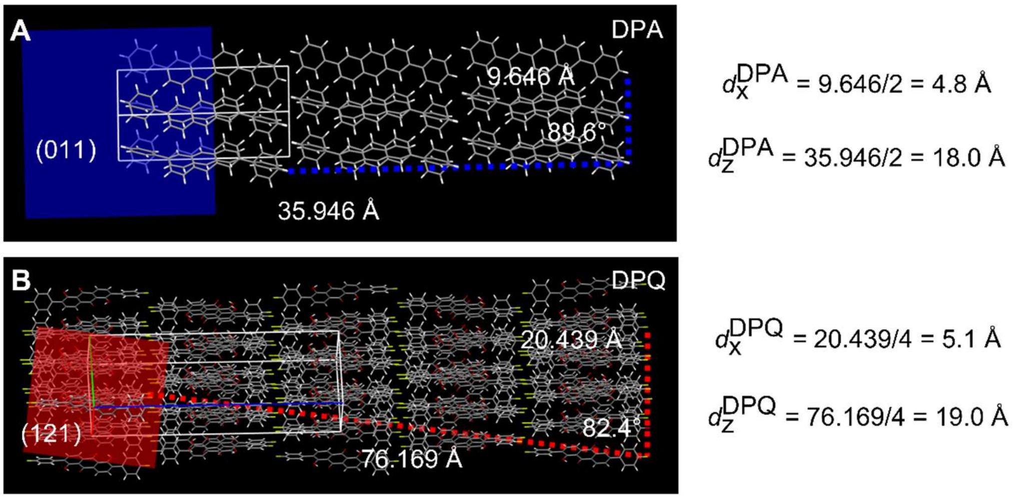


**Fig. S16. Calculate the adjacent intermolecular distance.**

(**A**) The adjacent intermolecular distance of DPA crystals along the (011) plane calculated by Mercury software is 4.8 Å and 18.0 Å, respectively. (**B**) The adjacent intermolecular distance of DPQ crystals along the (121) plane calculated by Mercury software is 5.1 Å and 19.0 Å, respectively.

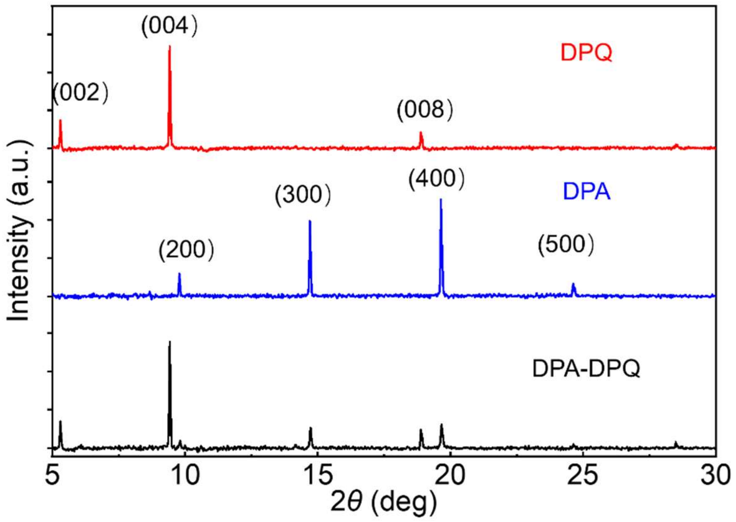


**Fig. S17. Structural characterization.**

X-ray diffraction patterns (XRD) of the DPQ microcrystals (red line), DPA microcrystals (blue line), and DPA-DPQ 2D OLHs (black line).

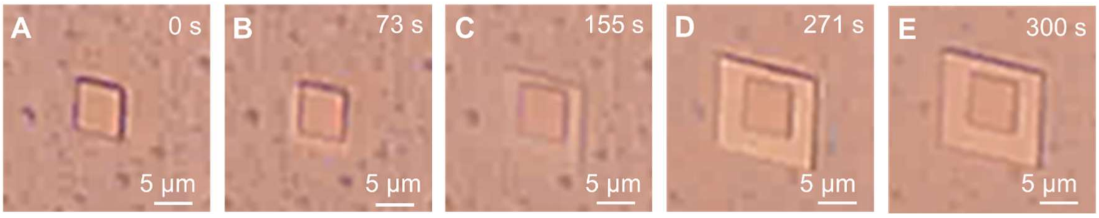


**Fig. S18. Heterostructure growth process.**

The evolution processes of DPA-DPQ 2D OLHs at different time intervals (**A**, 0 s; **B**, 73 s; **C**, 155 s; **D**, 271 s; **E**, 300 s) were recorded by the corresponding real-time video.

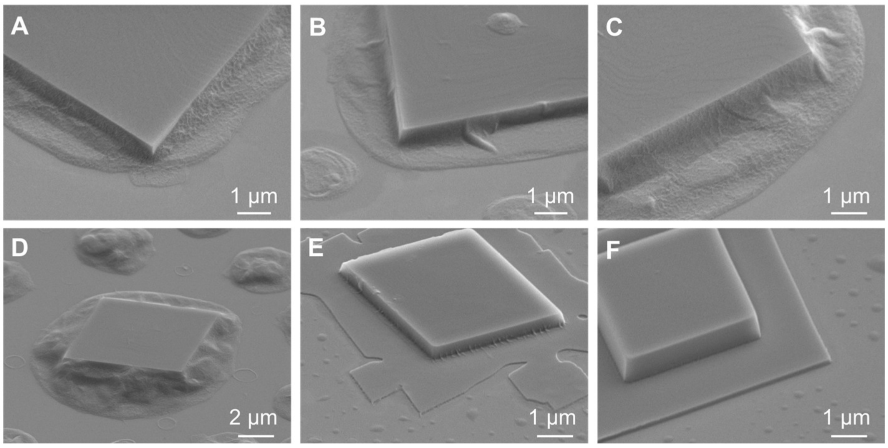


**Fig. S19. Morphology characterization.**

(**A**-**F**) SEM images of the transition state during the growth process of DPA-DPQ 2D OLHs.

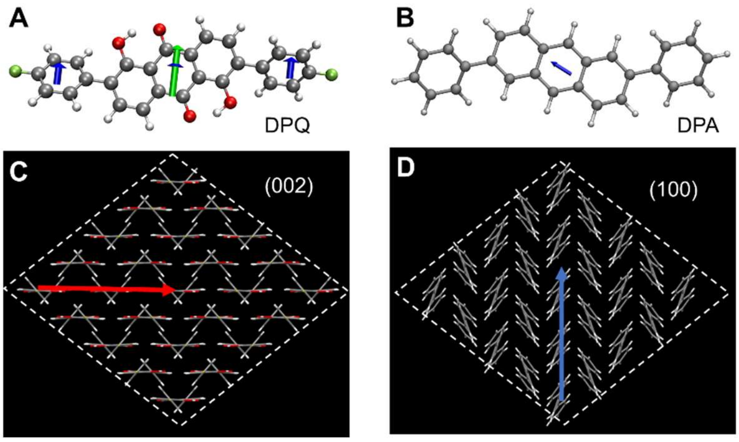


**Fig. S20. Calculation of the transition dipole moment (TDM).**

Simulated structure of the DPQ (**A**) and DPA (**B**) molecule and the direction of the calculated TDM of the DPQ and DPA molecule (direction pointed by the green and blue arrow). (**C**) Molecular arrangement simulation of the DPQ (002) crystal plane, with the direction of TDM is pointed by a red arrow. (**D**) Molecular arrangement simulation of the DPA (100) crystal plane, with the direction of TDM is pointed by a blue arrow.

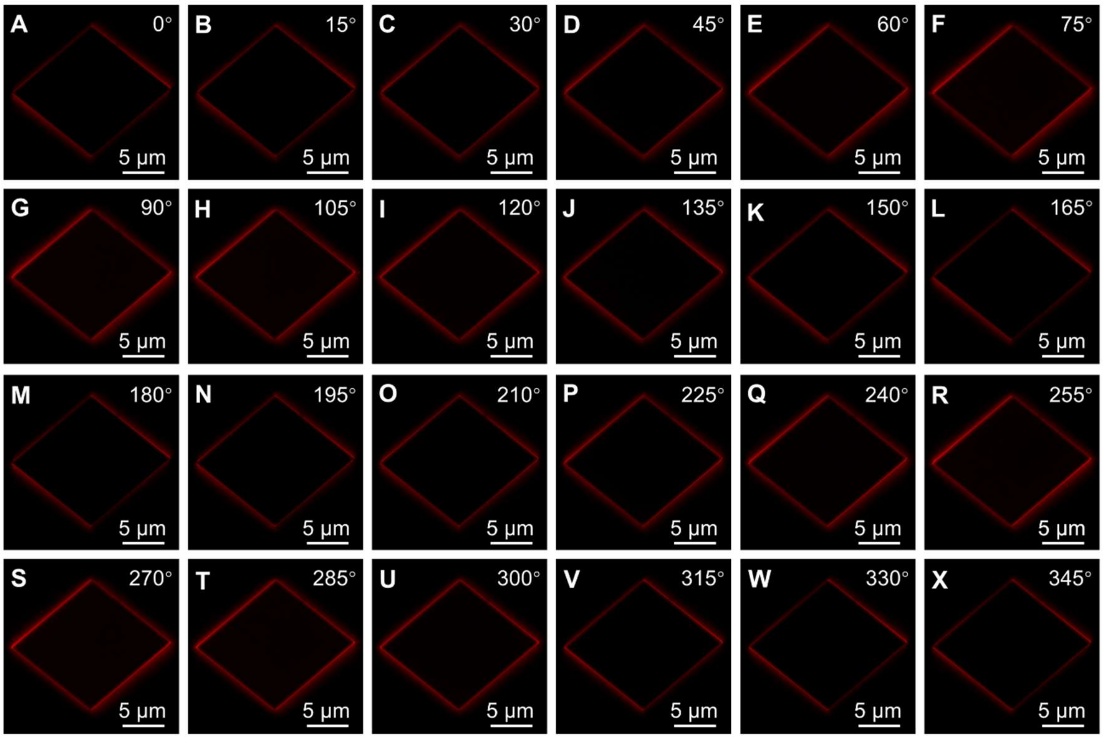


**Fig. S21. Polarization analysis of DPQ microcrystal.**

(**A-X**) FM images of a DPQ microcrystal at different polarization angles.

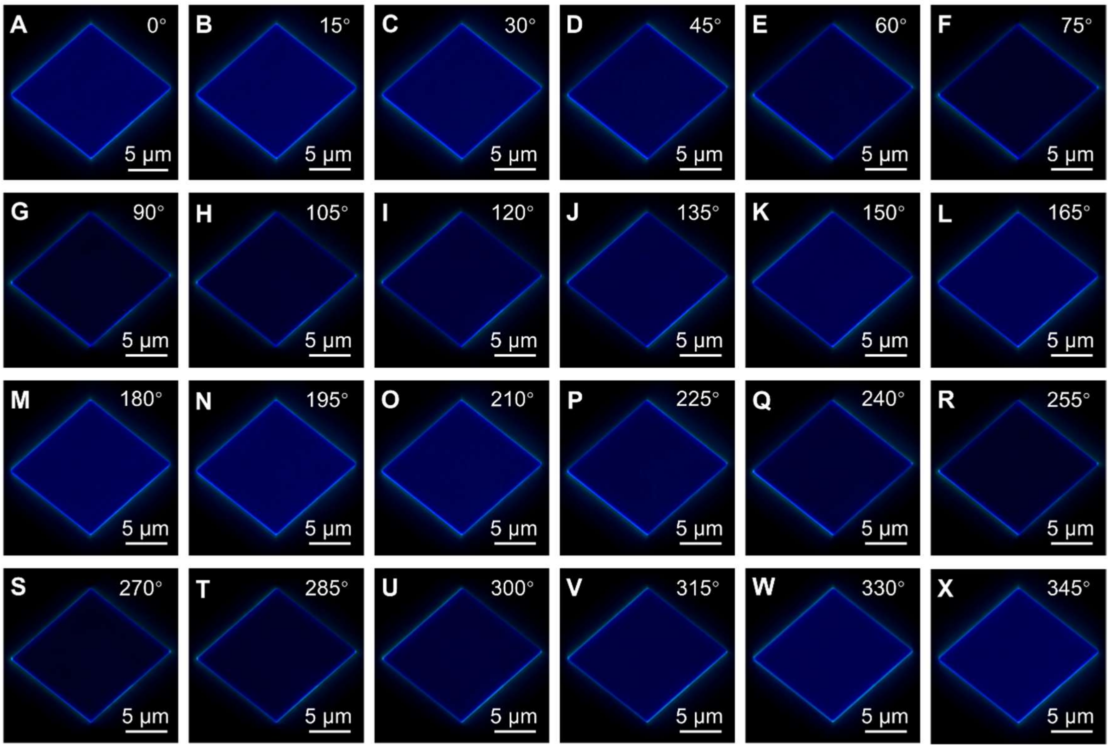


**Fig. S22. Polarization analysis of DPA microcrystal.**

(**A**-**X**) FM images of a DPA microcrystal at different polarization angles.

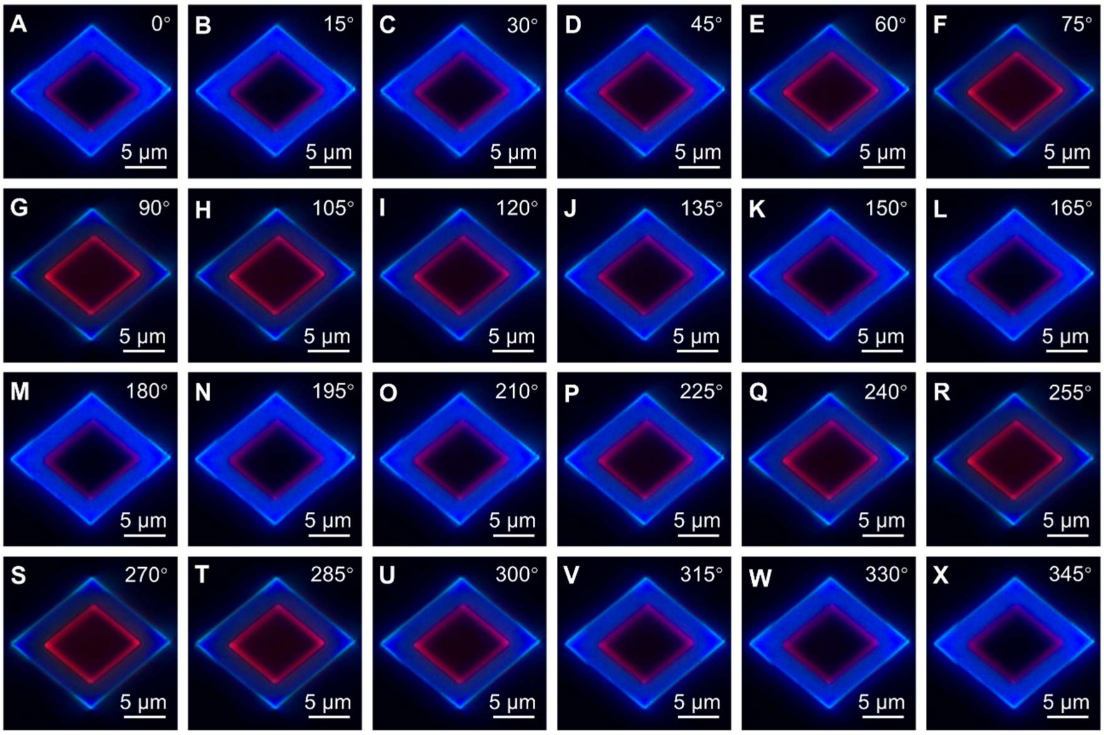


**Fig. S23**. **Polarization analysis of DPA-DPQ heterostructure.**

(**A**-**X**) FM images of a DPA-DPQ heterostructure at different polarization angles.

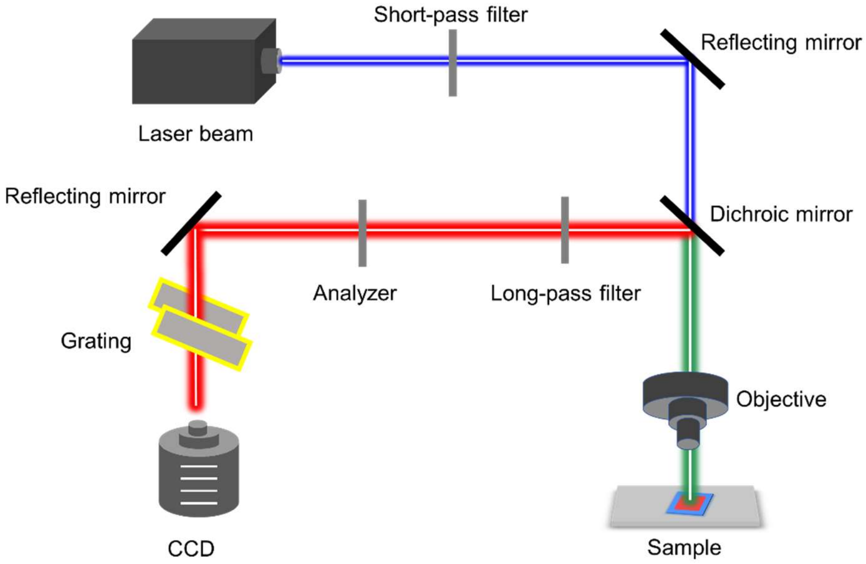


**Fig. S24. Experimental setup for optical characterization.**

Schematic demonstration of a homemade optical microscopy experimental setup for optical characterization.

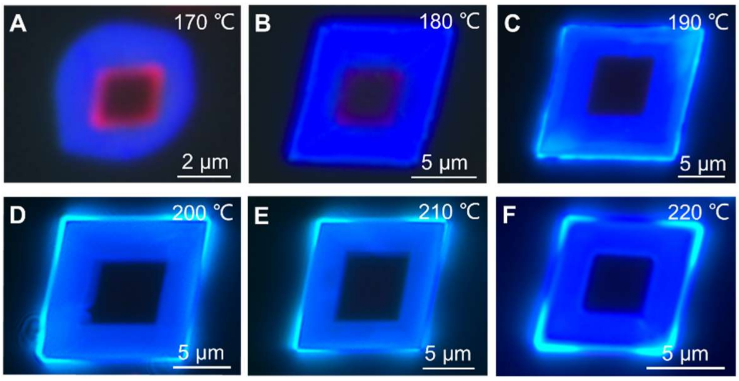


**Fig. S25. FM images of the OLHs prepared at different DPA sublimation temperatures.**
FM images of the DPA-DPQ 2D OLHs obtained at different DPA sublimation temperatures, 170 °C (**A**), 180 °C (**B**), 190 °C (**C**), 200 °C (**D**), 210 °C (**E**), and 220 °C (**F**), respectively.

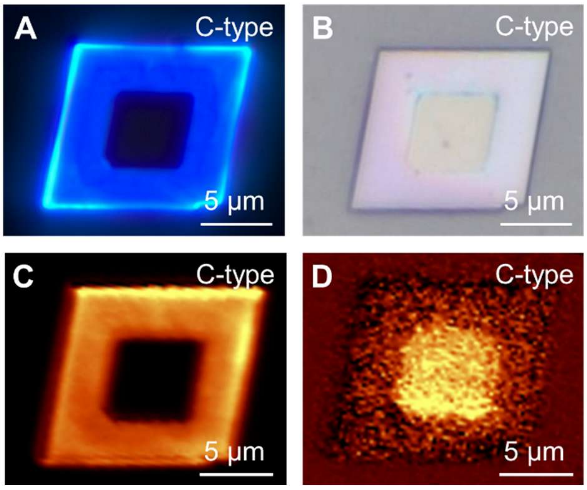


**Fig. S26. Optical properties of the C-type OLHs.**

FM (**A**) and bright-field microscope (**B**) images of a single C-type DPA-DPQ heterostructure. (**C**) PL map of the C-type heterostructure at 450 nm (blue area signal). (**D**) PL map of the C-type heterostructure at 670 nm (red area signal).

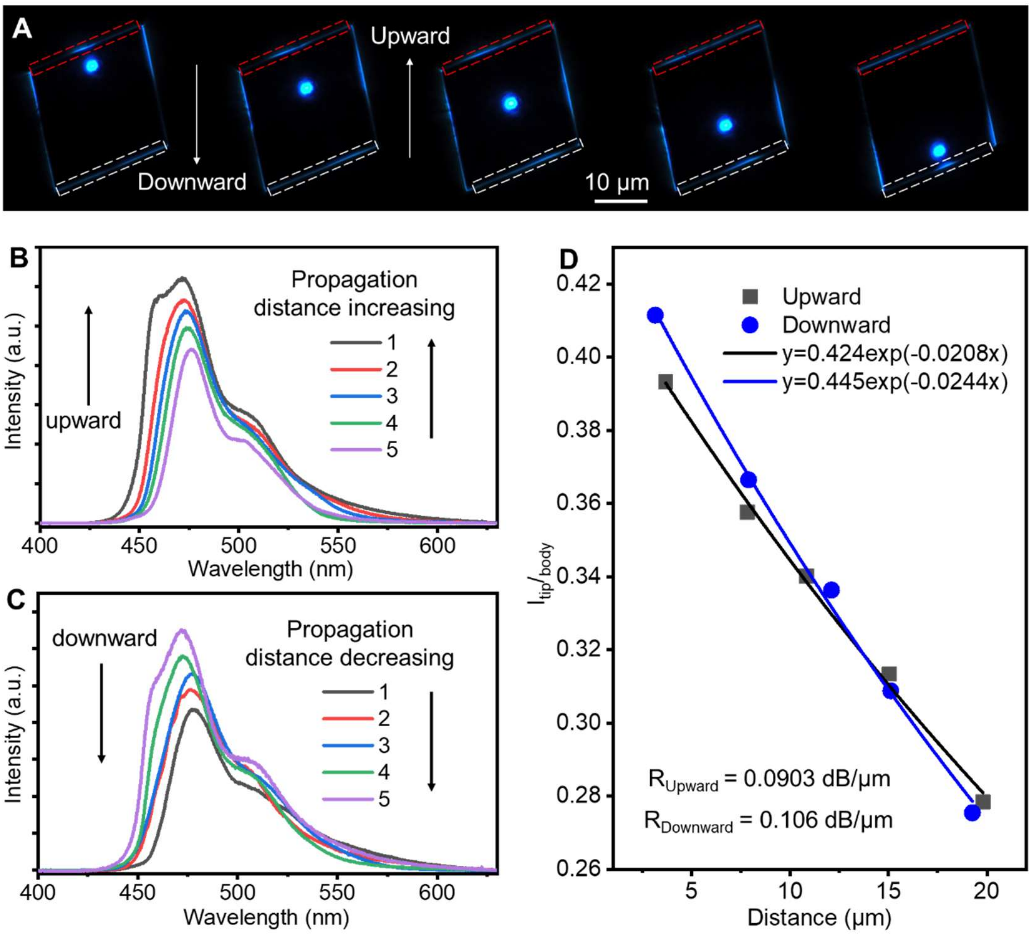


**Fig. S27. Optical waveguide property of the DPA microcrystal.**

(**A**) FM images were obtained from an individual DPA microcrystal by exciting with a laser beam ($\lambda = 375$ nm) at different positions along the upward and downward directions. The corresponding spatially resolved PL spectra in (**A**) for the upward (**B**) and the downward (**C**) directions with different separation distances. (**D**) The ratios of the intensity $I_{tip}/I_{body}$ as a function of the distance $d$ for (**A**). Curves were fitted by an exponential decay function $I_{tip}/I_{body} = A\exp(-RD)$.

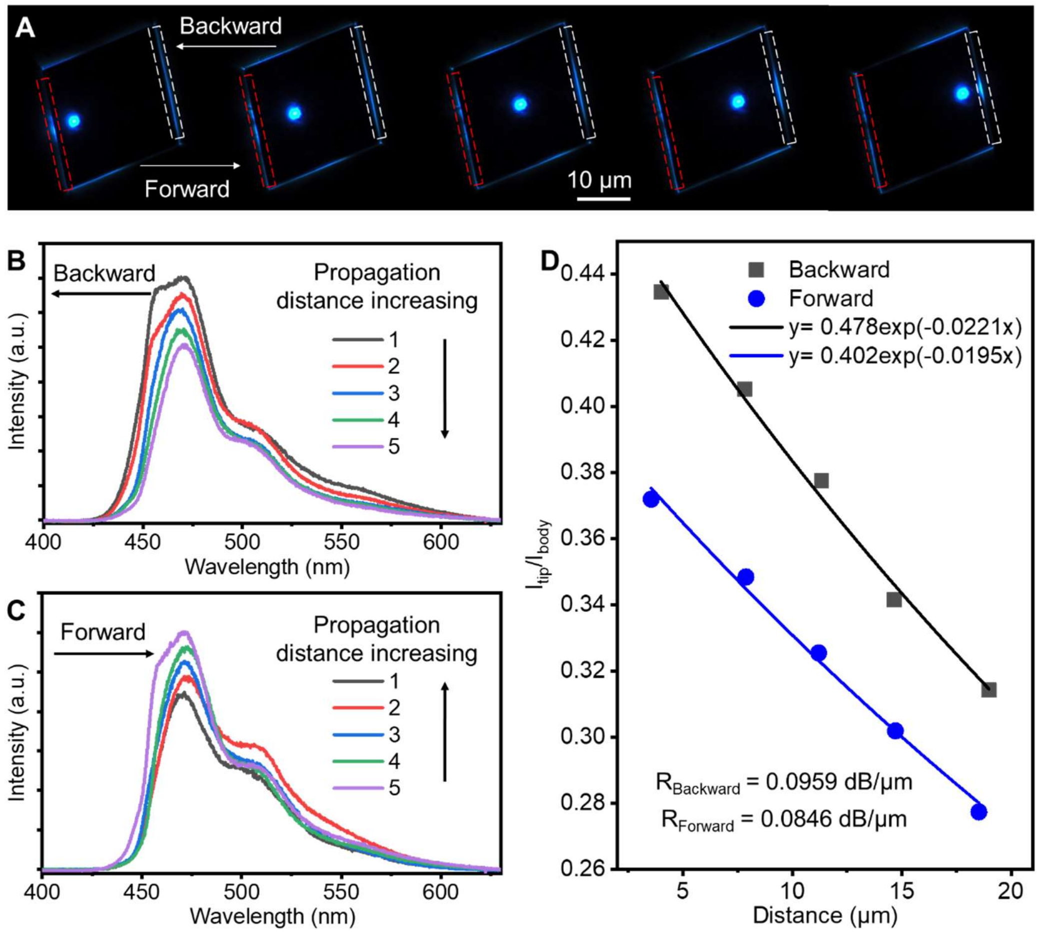


**Fig. S28. Optical waveguide property of the DPA microcrystal.**

(**A**) FM images were obtained from an individual DPA microcrystal by exciting with a laser beam ($\lambda$ = 375 nm) at different positions along the backward and forward directions. The corresponding spatially resolved PL spectra in (**A**) for the backward (**B**) and forward (**C**) directions with different separation distances. (**D**) The ratios of the intensity $I_{tip}/I_{body}$ as a function of the distance $d$ for (**A**). Curves were fitted by an exponential decay function $I_{tip}/I_{body} = A\exp(-RD)$.

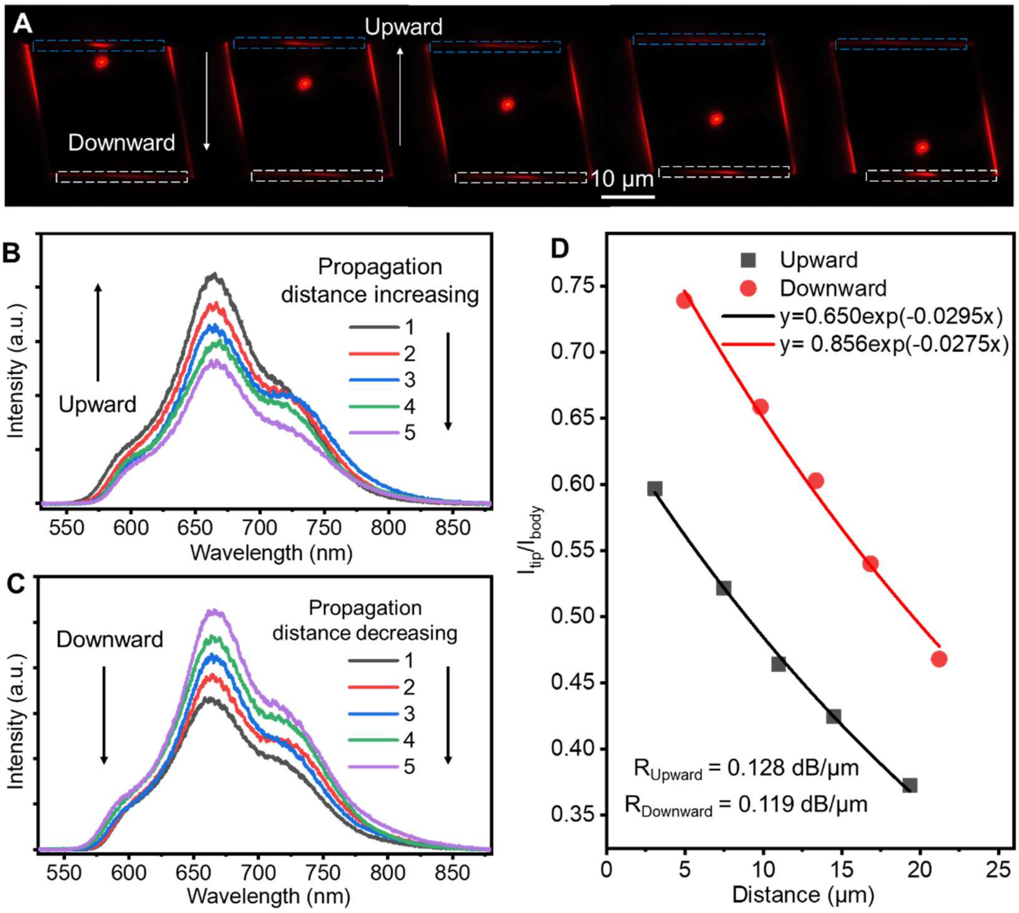


**Fig. S29. Optical waveguide property of the DPQ microcrystal.**

(**A**) FM images were obtained from an individual DPQ microcrystal by exciting with a laser beam ($\lambda$ = 375 nm) at different positions along the upward and downward directions. The corresponding spatially resolved PL spectra in (**A**) for the upward (**B**) and the downward (**C**) directions with different separation distances. (**D**) The ratios of the intensity $I_{tip}/I_{body}$ as a function of the distance $d$ for (**A**). Curves were fitted by an exponential decay function $I_{tip}/I_{body} = A\exp(-RD)$.

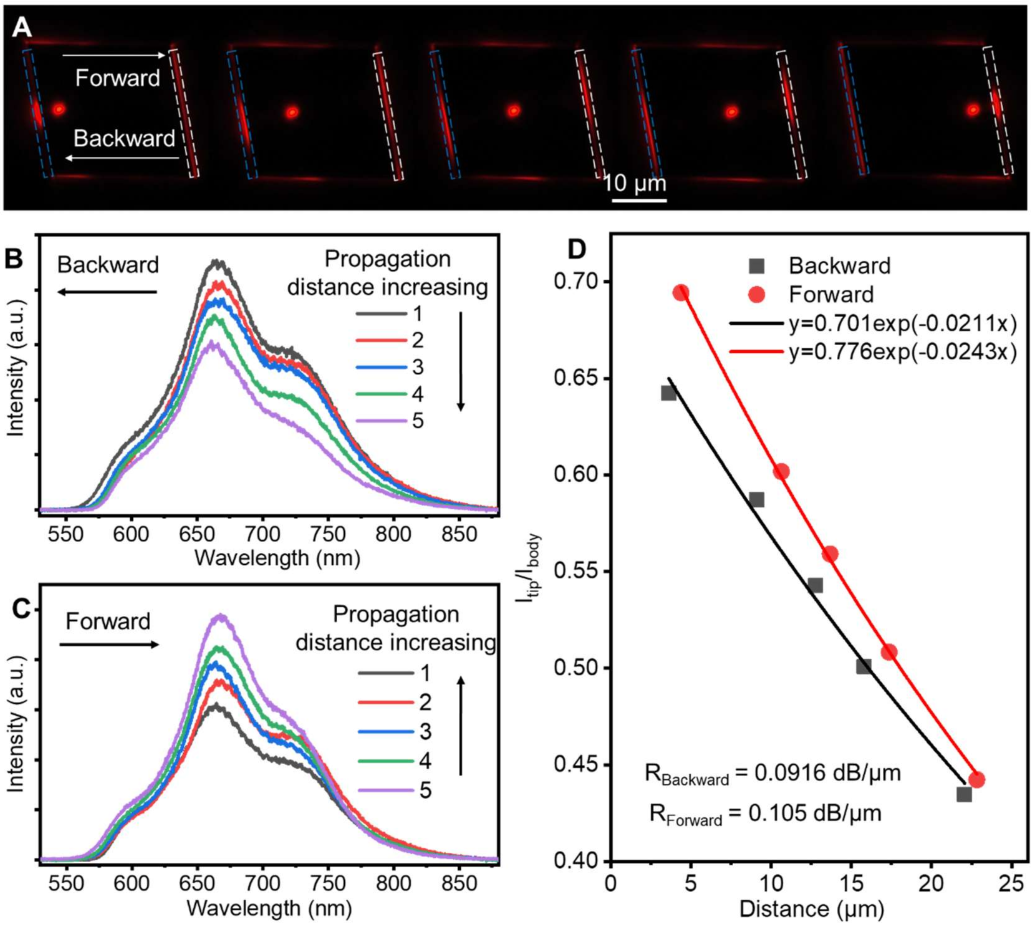


**Fig. S30. Optical waveguide property of the DPQ microcrystal.**

(**A**) FM images were obtained from an individual DPQ microcrystal by exciting with a laser beam ($\lambda$ = 375 nm) at different positions along the backward and forward directions. The corresponding spatially resolved PL spectra in (**A**) for the backward (**B**) and forward (**C**) directions with different separation distances. (**D**) The ratios of the intensity $I_{tip}/I_{body}$ as a function of the distance $d$ for (**A**). Curves were fitted by an exponential decay function $I_{tip}/I_{body} = A\exp(-RD)$.

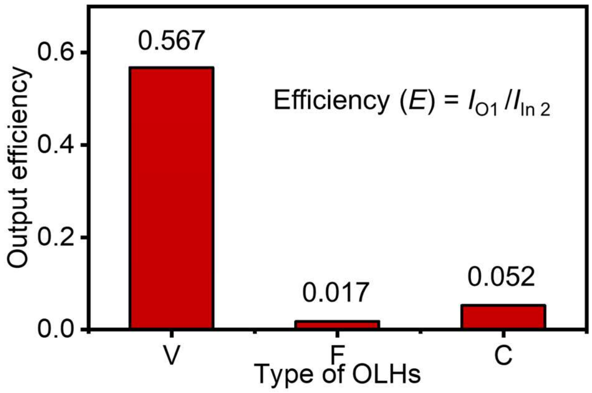


**Fig. S31. Output efficiency analysis of O1 position.**

Plot of output efficiency statistics for the O1 position in V-type, F-type, and C-type OLHs.

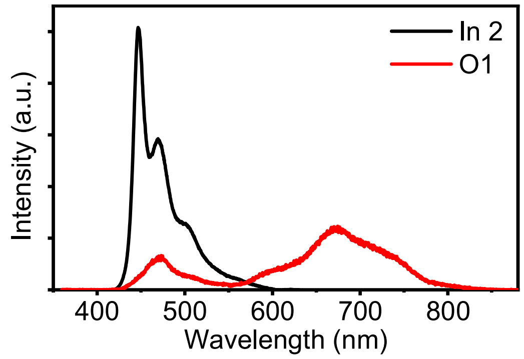


**Fig. S32. PL spectra at different positions of the V-type heterostructure.**

The input signal was collected at the In 2 position, and the output signal was collected at the O1 position of the V-type heterostructure in Fig. 5**J**.

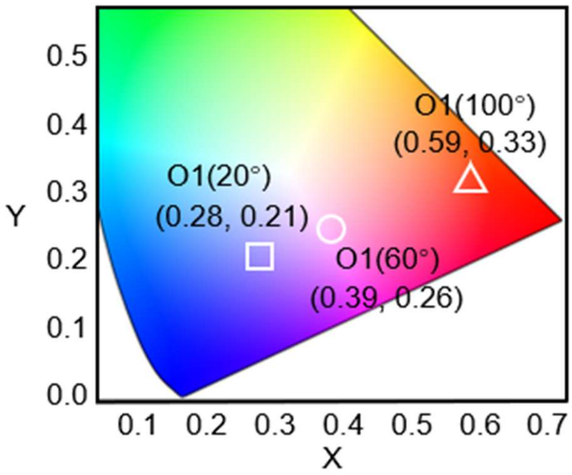


**Fig. S33. CIE chromaticity coordinates.**

Calculation of CIE chromaticity coordinates at O1 at polarization angles of 20°, 60°, and 100° using spatially resolved PL spectra.

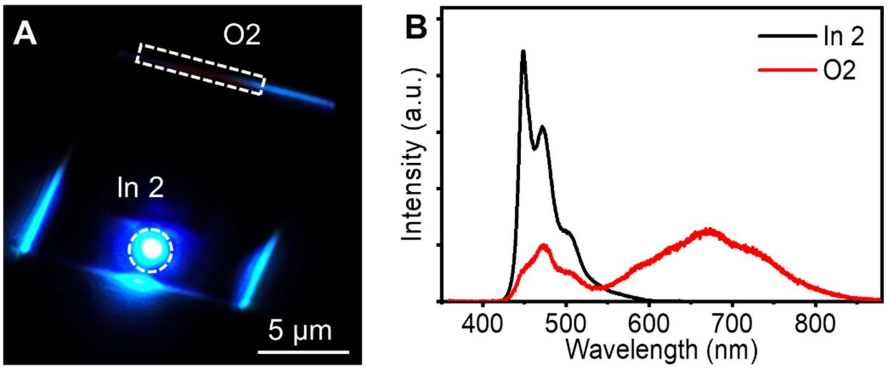


**Fig. S34. Optical waveguide analysis of F-type heterostructure.**

(**A**) FM image of a 405 nm laser beam directed at the edge position of the F-type heterostructure. In 2, light input excitation spot (white dashed circle). O2, light output edge (white dashed rectangle). (**B**) The input signal was collected at the In 2 position, and the output signal was collected at the O2 position of the F-type heterostructure.

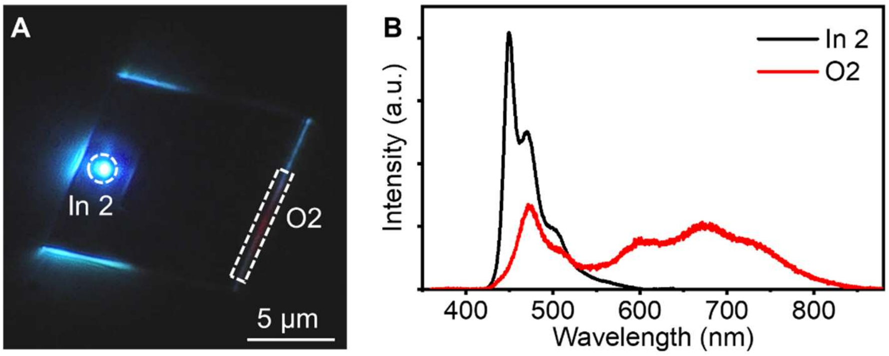


**Fig. S35. Optical waveguide analysis of C-type heterostructure.**

(**A**) FM image of a 405 nm laser beam directed at the edge position of the C-type heterostructure. In 2, light input excitation spot (white dashed circle). O2, light output edge (white dashed rectangle). (**B**) The input signal was collected at the In 2 position, and the output signal was collected at the O2 position of the C-type heterostructure.

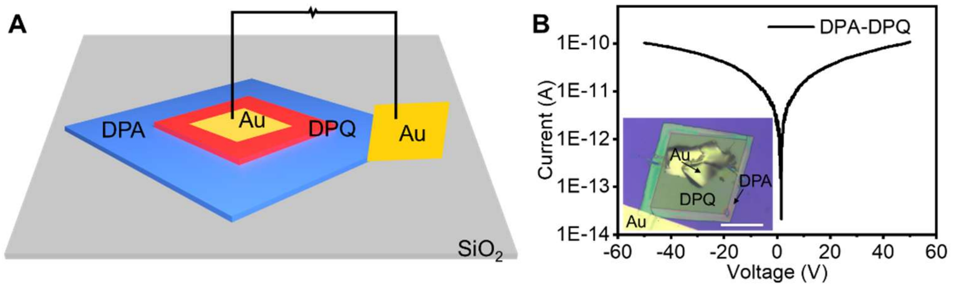


**Fig S36. *I-V* curve of the DPA-DPQ heterostructures.**

(**A**) Schematic device structure of the DPA-DPQ lateral heterojunction. (**B**) *I-V* curves of the DPA-DPQ lateral heterojunction. (Inset: bright field micrograph image of the device with a scale bar of 10 μm).

**Table S1. The summary of the parameters of the DPA (CCDC 1044209) and DPQ (CCDC-2435358) unit cell.**

| Name | DPA | DPQ |
|---|---|---|
| Formula | $C_{26}H_{18}$ | $C_{26}H_{14}F_2O_4$ |
| Formula Weight | 330.42 | 428.37 |
| Crystal system | Monoclinic | Orthorhombic |
| Space group | P $2_1$/c | Ibca |
| | $a$ = 17.973(8) | $a$ = 7.9208(3) |
| cell Lengths(Å) | $b$ = 7.352(3) | $b$ = 12.9146(5) |
| | $c$ = 6.245(3) | $c$ = 37.5331(17) |
| Cell Angles(°) | $\alpha$ = 90 $\beta$ = 90.646(9) $\gamma$ = 90 | $\alpha$ = 90 $\beta$ = 90 $\gamma$ = 90 |
| Cell Volume ($Å^3$) | 825.1 | 3839.4 |
| Z, Z' | Z: 2 Z': 0 | Z: 8 Z': 0.5 |
| R-Factor (%) | 5.63 | 3.99 |

**Table S2. Calculated attachment energies of different crystal facets of the DPA crystal by using the Material Studio simulation.**

| {hkl} | $d_{\{hkl\}}$(Å) | $E_{att}$(kcal/mol) | % Total facet area |
|---|---|---|---|
| {100} | 17.97 | -15.98 | 65.44 |
| {110} | 6.80 | -55.62 | 13.20 |
| {011} | 4.76 | -69.28 | 21.36 |

**Table S3. Calculated attachment energies of different crystal facets of the DPQ crystal by using the Material Studio simulation.**

| {hkl} | $d_{\{hkl\}}$(Å) | $E_{att}$(kcal/mol) | % Total facet area |
|---|---|---|---|
| {002} | 18.77 | -40.76 | 71.55 |
| {020} | 6.46 | -208.75 | 7.19 |
| {112} | 6.35 | -218.16 | 13.50 |
| {121} | 4.96 | -222.99 | 7.76 |

**Table S4. Calculated surface energies of different crystal facets of the DPA crystal by using the Material Studio simulation.**

| {hkl} | $d_{\{hkl\}}$(Å) | $E_{surf}$(kcal/mol/Å$^2$) | % Total facet area |
|---|---|---|---|
| {100} | 17.97 | 0.174 | 33.00 |
| {110} | 6.80 | 0.240 | 0.76 |
| {011} | 4.76 | 0.208 | 22.42 |
| {11-1} | 4.61 | 0.209 | 17.48 |
| {111} | 4.59 | 0.240 | 1.66 |
| {21-1} | 4.22 | 0.230 | 1.17 |
| {020} | 3.68 | 0.208 | 7.61 |
| {120} | 3.60 | 0.221 | 7.08 |

**Table S5. Calculated surface energies of different crystal facets of DPQ crystal by using the Material Studio simulation.**

| {hkl} | $d_{\{hkl\}}$(Å) | $E_{att}$(kcal/mol) | % Total facet area |
|---|---|---|---|
| {002} | 18.77 | 0.100 | 40.04 |
| {020} | 6.46 | 0.178 | 1.43 |
| {121} | 4.96 | 0.150 | 43.64 |
| {123} | 4.65 | 0.163 | 4.00 |
| {200} | 3.96 | 0.192 | 1.98 |
| {202} | 3.88 | 0.197 | 1.52 |

## REFERENCES


1. J. Tong, Y. Fu, D. Domaretskiy, F. Della Pia, P. Dagar, L. Powell, D. Bahamon, S. Huang, B. Xin, R. N. Costa Filho, L. F. Vega, I. V. Grigorieva, F. M. Peeters, A. Michaelides, M. Lozada-Hidalgo, Control of proton transport and hydrogenation in double-gated graphene. *Nature* **630**, 619–624 (2024).

2. J. Zhang, F. Wang, V. B. Shenoy, M. Tang, J. Lou, Towards controlled synthesis of 2D crystals by chemical vapor deposition (CVD). *Mater. Today* **40**, 132–139 (2020).

3. C. Schönenberger, 2D materials shrink superconducting qubits. *Nat. Mater.* **21**, 381–382 (2022).

4. J. Zhang, B. Tan, X. Zhang, F. Gao, Y. Hu, L. Wang, X. Duan, Z. Yang, P. A. Hu, Atomically thin hexagonal boron nitride and its heterostructures. *Adv. Mater.* **33**, e2000769 (2021).

5. N. Stingelin-Stutzmann, E. Smits, H. Wondergem, C. Tanase, P. Blom, P. Smith, D. de Leeuw, Organic thin-film electronics from vitreous solution-processed rubrene hypereutectics. *Nat. Mater.* **4**, 601–606 (2005).

6. K. S. Kim, D. Lee, C. S. Chang, S. Seo, Y. Hu, S. Cha, H. Kim, J. Shin, J.-H. Lee, S. Lee, J. S. Kim, K. H. Kim, J. M. Suh, Y. Meng, B.-I. Park, J.-H. Lee, H.-S. Park, H.-S. Kum, M.-H. Jo, G. Y. Yeom, K. Cho, J.-H. Park, S.-H. Bae, J. Kim, Non-epitaxial single-crystal 2D material growth by geometric confinement. *Nature* **614**, 88–94 (2023).

7. Y. Zhao, C. Zhang, D. Kohler, J. M. Scheeler, J. C. Wright, P. M. Voyles, S. Jin, Supertwisted spirals of layered materials enabled by growth on non-Euclidean surfaces. *Science* **370**, 442–445 (2020).

8. J. Yuan, C. Jian, Z. Shang, Y. Yao, B. Wang, Y. Li, R. Wang, Z. Fu, M. Li, W. Hong, X. He, Q. Cai, W. Liu, Controllable synthesis of nonlayered high-κ $Mn_3O_4$ single-crystal thin films for 2D electronics. *Nat. Commun.* **16**, 964 (2025).

9. C. Wan, H. Dong, L. Jiang, W. Hu, Organic semiconductor crystals. *Chem. Soc. Rev.* **47**, 422–500 (2018).

10. C. Liao, Y. Gong, Y. Che, H. Ji, B. Liu, L. Zang, Y. Che, J. Zhao, Concentric hollow multi-hexagonal platelets from a small molecule. *Nat. Commun.* **15**, 5668 (2024).

11. J. Sun, Y. Choi, Y. J. Choi, S. Kim, J.-H. Park, S. Lee, J. H. Cho, 2D-organic hybrid heterostructures for optoelectronic applications. *Adv. Mater.* **31**, e1803831 (2019).

12. Y. Gong, L. Fu, Y. Che, H. Ji, Y. Zhang, L. Zang, J. Zhao, Y. Che, Fabrication of two-dimensional platelets with heat-resistant luminescence and large two-photon absorption cross sections via cooperative solution/solid self-assembly. *J. Am. Chem. Soc.* **145**, 9771–9776 (2023).

13. L. Lan, L. Li, C. Wang, P. Naumov, H. Zhang, Efficient aerial water harvesting with self-sensing dynamic janus crystals. *J. Am. Chem. Soc.* **146**, 30529–30538 (2024).

14. M.-P. Zhuo, Y.-C. Tao, X.-D. Wang, Y. Wu, S. Chen, L.-S. Liao, L. Jiang, 2D organic photonics: An asymmetric optical waveguide in self-assembled halogen-bonded cocrystals. *Angew. Chem. Int. Ed. Engl.* **130**, 11470–11474 (2018).

15. L. Zhang, M. Hasan, Y. Tang, A. R. Khan, H. Yan, T. Yildirim, X. Sun, J. Zhang, J. Zhu, Y. Zhang, Y. Lu, 2D organic single crystals: Synthesis, novel physics, high-performance optoelectronic devices and integration. *Mater. Today* **50**, 442–475 (2021).

16. H. Dong, C. Zhang, F.-J. Shu, C.-L. Zhou, Y. Yan, J. Yao, Y. Zhao, Superkinetic growth of oval organic semiconductor microcrystals for chaotic lasing. *Adv. Mater.* **33**, e2100484 (2021).

17. T. Zhang, Z. Zhou, X. Liu, K. Wang, Y. Fan, C. Zhang, J. Yao, Y. Yao, Y. S. Zhao, Thermally activated lasing in organic microcrystals toward laser displays. *J. Am. Chem. Soc.* **143**, 20249–20255 (2021).

18. K. Wang, Y. S. Zhao, Pursuing electrically pumped lasing with organic semiconductors. *Chem* **7**, 3221–3231 (2021).

19. V. V. Pradeep, R. Chandrasekar, Micromanufacturing of geometrically and dimensionally precise molecular single-crystal photonic microresonators via focused ion beam milling. *Adv. Opt. Mater.* **10**, 2201150 (2022).

20. V. V. Pradeep, N. Mitetelo, M. Annadhasan, M. Popov, E. Mamonov, T. Murzina, R. Chandrasekar, Ambient pressure sublimation technique provides polymorph-selective perylene nonlinear optical microcavities. *Adv. Opt. Mater.* **8**, 1901317 (2020).

21. F. Yang, S. Cheng, X. Zhang, X. Ren, R. Li, H. Dong, W. Hu, 2D organic materials for optoelectronic applications. *Adv. Mater.* **30**, 1702415 (2018).

22. M. Annadhasan, D. P. Karothu, R. Chinnasamy, L. Catalano, E. Ahmed, S. Ghost, P. Naumov, R. Chandrasekar, Micromanipulation of mechanically compliant organic single-crystal optical microwaveguides. *Angew. Chem. Int. Ed. Engl.* **132**, 13925–13934 (2020).

23. Z. Qin, T. Wang, H. Cao, Y. Li, H. Dong, W. Hu, Organic polarized light-emitting transistors. *Adv. Mater.* **35**, e2301955 (2023).

24. A Khapre, A. V. Kumar, R. Chandrasekar, Powering organic flexible optical waveguides and circuits via focused micro-LEDs for visible light communication. *Laser Photonics Rev.* **19**, 2400278 (2025).

25. A. Khapre, J. Hazarika, R. Chandrasekar, Organic crystal active waveguide as an all-angle signal receiver and transmission platform for visible light communication. arXiv:2506.04874 (2025).

26. A. K. Geim, I. V. Grigorieva, Van der Waals heterostructures. *Nature* **499**, 419–425 (2013).

27. S. Yang, B. Xu, X. Feng, R. Lin, Y. Xu, S. Chen, Z. Wang, X. Wang, X. Meng, Z. Gao, Laterally engineering organic light-harvesting monolithic heterostructures for spatially resolved photonic barcodes. *Chem. Mater.* **35**, 7094–7102 (2023).

28. Z.-J. Lv, Q. Lv, T.-Z. Feng, J.-H. Jiang, X.-D. Wang, Epitaxial growth of two-dimensional organic crystals with in-plane heterostructured domain regulation. *J. Am. Chem. Soc.* **146**, 25755–25763 (2024).

29. Y. Shi, L. Jiang, J. Liu, Z. Tu, Y. Hu, Q. Wu, Y. Yi, E. Gann, C. R. McNeill, H. Li, W. Hu, D. Zhu, H. Sirringhaus, Bottom-up growth of n-type monolayer molecular crystals on polymeric substrate for optoelectronic device applications. *Nat. Commun.* **9**, 2933 (2018).

30. Q. Lv, X.-D. Wang, Y. Yu, C.-F. Xu, Y.-J. Yu, X.-Y. Xia, M. Zheng, L.-S. Liao, Lateral epitaxial growth of two-dimensional organic heterostructures. *Nat. Chem.* **16**, 201–209 (2024).

31. T.-Z. Feng, Q. Lv, Z.-J. Lv, C.-F. Xu, J.-H. Jiang, X.-D. Wang, In-plane hierarchical assembly of two-dimensional molecular crystals toward on-chip multimode optical waveguides. *Sci. Adv.* **11**, eadt0938 (2025).

32. X. Ye, Y. Liu, Q. Han, C. Ge, S. Cui, L. Zhang, X. Zheng, G. Liu, J. Liu, D. Liu, X. Tao, Microspacing in-air sublimation growth of organic crystals. *Chem. Mater.* **30**, 412–420 (2018).

33. S. Zhao, J.-X. Zhang, C.-F. Xu, Y. Ma, J.-H. Luo, H. Lin, Y. Shi, X.-D. Wang, L.-S. Liao, Programmable in-situ co-assembly of organic multi-block nanowires for cascade optical waveguides. *Angew. Chem. Int. Ed. Engl.* **63**, e202412712 (2024).

34. B. Wu, M. Zheng, M.-P. Zhuo, Y.-D. Zhao, Y. Su, J.-Z. Fan, P. Luo, L.-F. Gu, Z.-L. Che, Z.-S. Wang, X.-D. Wang, Organic bilayer heterostructures with built-in exciton conversion for 2D photonic encryption. *Adv. Mater.* **35**, e2306541 (2023).

35. T. Lu, A comprehensive electron wavefunction analysis toolbox for chemists, Multiwfn. *J. Chem. Phys.* **161**, 082503 (2024).

36. T. Lu, F. Chen, Multiwfn: A multifunctional wavefunction analyzer. *J. Comput. Chem.* **33**, 580–592 (2012).

37. J. Valenta, I. Pelant, K. Luterova, R. Tomasiunas, S. Cheylan, R. G. Ellima, J. Linnros, B. Honerlage, Active planar optical waveguide made from luminescent silicon nanocrystals. *Appl. Phys. Lett.* **82**, 955–957 (2023).

38. N. Chandrasekhar, A. Mohiddon, R. Chandrasekar, Organic submicro tubular optical waveguides: Self-assembly, diverse geometries, efficiency, and remote sensing properties. *Adv. Opt. Mater.* **1**, 305–311 (2013).

39. W. Zhu, L. Zhu, L. Sun, Y. Zhen, H. Dong, Z. Wei, W. Hu, Uncovering the intramolecular emission and tuning the nonlinear optical properties of organic materials by cocrystallization. *Angew. Chem. Int. Ed. Engl.* **55**, 14023–14027 (2016).
